# A new role for histone demethylases in the maintenance of plant genome integrity

**DOI:** 10.7554/eLife.58533

**Published:** 2020-10-27

**Authors:** Javier Antunez-Sanchez, Matthew Naish, Juan Sebastian Ramirez-Prado, Sho Ohno, Ying Huang, Alexander Dawson, Korawit Opassathian, Deborah Manza-Mianza, Federico Ariel, Cecile Raynaud, Anjar Wibowo, Josquin Daron, Minako Ueda, David Latrasse, R Keith Slotkin, Detlef Weigel, Moussa Benhamed, Jose Gutierrez-Marcos

**Affiliations:** 1 School of Life Science, University of WarwickCoventryUnited Kingdom; 2 Université Paris-Saclay, CNRS, INRAE, Univ Evry, Institute of Plant Sciences Paris-Saclay (IPS2)OrsayFrance; 3 Graduate School of Agriculture, Kyoto University, Kitashirakawa Oiwake-cho, Sakyo-kuKyotoJapan; 4 Department of Molecular Biology, Max Planck Institute for Developmental BiologyTübingenGermany; 5 Department of Molecular Genetics, The Ohio State UniversityColumbusUnited States; 6 Institute of Transformative Bio-Molecules, Nagoya UniversityNagoyaJapan; 7 Division of Biological Science, Graduate School of Science, Nagoya UniversityNagoyaJapan; 8 Donald Danforth Plant Science CenterSt. LouisUnited States; 9 Division of Biological Sciences, University of MissouriColumbiaUnited States; 10 Université de Paris, Institute of Plant Sciences Paris-Saclay (IPS2), F-75006ParisFrance; University of LausanneSwitzerland; Seoul National UniversityRepublic of Korea

**Keywords:** chromatin, DNA methylation, epimutation, transposon, *A. thaliana*

## Abstract

Histone modifications deposited by the Polycomb repressive complex 2 (PRC2) play a critical role in the control of growth, development, and adaptation to environmental fluctuations of most multicellular eukaryotes. The catalytic activity of PRC2 is counteracted by Jumonji-type (JMJ) histone demethylases, which shapes the genomic distribution of H3K27me3. Here, we show that two JMJ histone demethylases in *Arabidopsis*, EARLY FLOWERING 6 (ELF6) and RELATIVE OF EARLY FLOWERING 6 (REF6), play distinct roles in H3K27me3 and H3K27me1 homeostasis. We show that failure to reset these chromatin marks during sexual reproduction results in the transgenerational inheritance of histone marks, which cause a loss of DNA methylation at heterochromatic loci and transposon activation. Thus, Jumonji-type histone demethylases play a dual role in plants by helping to maintain transcriptional states through development and safeguard genome integrity during sexual reproduction.

## Introduction

In eukaryotes, chromatin accessibility is modified by DNA methylation, the covalent modification of histone proteins and the deposition of histone variants. These epigenetic modifications allow the establishment of specific transcriptional states in response to environmental or developmental cues. While in most cases environmentally-induced chromatin changes are transient, epigenetic changes induced during development are often stably inherited through mitotic divisions, so that cell identity is maintained and individual cells or tissues do not revert to previous developmental states. A key chromatin modification implicated in these responses is the post-translational modification of histone tails, which are associated with active or inactive transcriptional states. Among these, the methylation of lysine 9 of histone H3 (H3K9me2) and H3K27me1 have been associated with the repression of transposable elements (TEs) in constitutive heterochromatin (Lindroth et al., 2004; Lippman et al., 2004; Mathieu et al., 2005), whereas other types of methylation, including H3K27me3, have been associated with the repression of genes in euchromatic genome regions (Berger, 2007; Pfluger and Wagner, 2007). H3K27me3 methylation is deposited by PRC2 and plays a crucial role in the development of most multicellular eukaryotes (Laugesen et al., 2019). In plants, this modification is found in approximately one quarter of protein-coding genes and is dynamically regulated during growth and development (Lafos et al., 2011; Roudier et al., 2011; Zhang et al., 2007). The activity of PRCs is counterbalanced by JMJ demethylases, which catalyze the specific removal of H3K27me3 (Liu et al., 2010). In *Arabidopsis*, five histone demethylases [RELATIVE OF EARLY FLOWERING 6 (REF6); EARLY FLOWERING 6 (ELF6); JUMONJI 13 (JMJ13); JUMONJI 30 (JMJ30); and JUMONJI 32 (JMJ32)] have been implicated in the demethylation of H3K27 (Crevillén et al., 2014; Gan et al., 2014; Lu et al., 2011). These proteins are thought to mediate the temporal and spatial de-repression of genes necessary for a wide range of plant processes such as flowering, hormone signaling, and control of the circadian clock. Inactivation of *REF6* results in the ectopic accumulation of H3K27me3 at hundreds of loci, many of them involved in developmental patterning and environmental responses (Lu et al., 2011; Yan et al., 2018). It has been proposed that REF6 is recruited to a specific sequence motif through its zinc-finger domain (Cui et al., 2016; Lu et al., 2011); however, others have shown that it is also recruited by specific interactions with transcription factors (Yan et al., 2018). Moreover, it has been shown that the affinity of REF6 to chromatin is hindered by DNA methylation, which could explain why its activity is primarily found at euchromatic loci (Qiu et al., 2019).

Previous studies have suggested that REF6 acts redundantly with ELF6 and JMJ13 to restrict the accumulation of H3K27me3 in gene regulatory regions, thereby unlocking tissue-specific expression (Yan et al., 2018). Importantly, REF6, ELF6, JMJ30 and JMJ32 appear to specifically remove methyl groups from H3K27me3 and H3K27me2 but not from H3K27me1 (Crevillén et al., 2014; Gan et al., 2014; Lu et al., 2011). Previous investigations have shown that H3K27me1 in *Arabidopsis* is associated with constitutive heterochromatin, where it is deposited by ARABIDOPSIS TRITHORAX-RELATED PROTEIN5 (ATXR5) and ATRX6 (Jacob et al., 2009; Jacob et al., 2010). However, several studies in mammals and plants have shown that H3K27me1 is also found in euchromatin (Fuchs et al., 2008; Jacob et al., 2009; Vakoc et al., 2006). The presence of H3K27me3 in euchromatin is thought to be actively re-set during sexual reproduction – a view supported by studies in *Arabidopsis* showing that ELF6, REF6 and JMJ13 are necessary to reset and prevent the inheritance of this epigenetic mark to the offspring (Crevillén et al., 2014; Liu et al., 2019; Zheng et al., 2019). However, the extent to which these epigenetic imprints are reset during sexual reproduction remains unknown.

Here, we show that the histone demethylases REF6 and ELF6 play distinct roles in the demethylation of histones in Arabidopsis, and that REF6 plays a major role in H3K27me1 dynamics in active chromatin. We also found that failure to reset H3K27me3 marks during sexual reproduction results in the inheritance of these epigenetic imprints even in the presence of fully functional histone demethylases. The ectopic inheritance of H3K27me3 is associated with the loss of DNA methylation at heterochromatic loci, leading to activation of TEs. Moreover, genetic and epigenetic mutations arising in histone demethylase mutants are stably inherited over multiple generations and result in pleiotropic developmental defects. Collectively, our work has uncovered a hitherto unrecognized role for histone demethylases in maintaining genetic and epigenetic stability of plant genomes.

## Results

### Arabidopsis REF6 and ELF6 play distinct roles in H3K27me3 homeostasis

**Figure 1.**
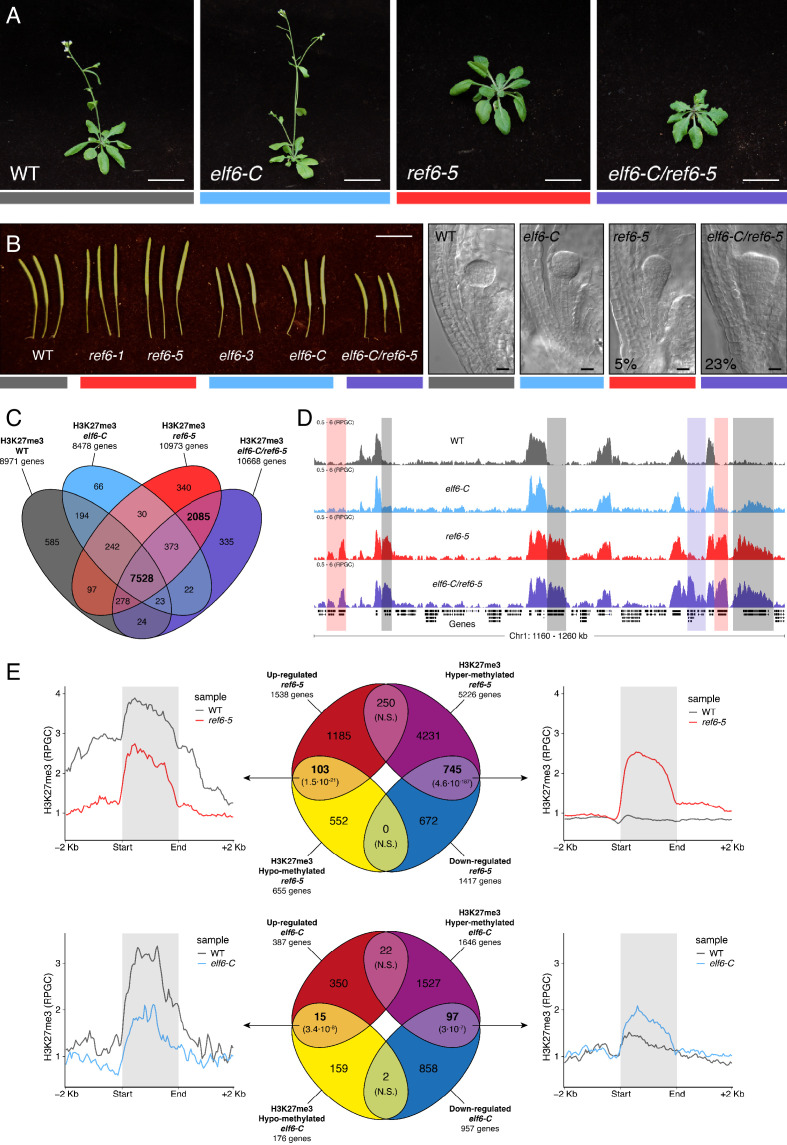
Arabidopsis Histone demethylases ELF6 and REF6 play distinct roles in development and H3K27me3 homeostasis. (**A**) Arabidopsis wild-type (WT) and histone demethylase mutants (*elf6-C*, *ref6-5* and *elf6-C*/*ref6-5*). Scale bars, 1 cm. (**B**) Siliques and embryos from Arabidopsis wild-type (WT) and different mutant alleles of histone demethylase ELF6 and REF6. Numbers show the frequency of the abnormal embryos (n = 250). Scale bars 1 cm and 10 μm, respectively. (**C**) Venn diagram showing the overlap between genes accumulating H3K27me3 in wild-type (WT) and histone demethylase mutants (*elf6-C*, *ref6-5* and *elf6-C*/*ref6-5*). (**D**) Genome browser views of background subtracted ChIP-seq signals as normalized reads per genomic content (RPGC). Shaded red boxes, genes targeted exclusively by REF6. Shaded grey boxes, genes targeted by REF6 and ELF6. Shaded purple boxes, genes targeted by both REF6 and ELF6, and only hyper-methylated in double mutant *elf6-C/ref6-5.* (**E**) Venn diagram showing overlap between differentially expressed genes (DEGs) and H3K27me3 differentially methylated genes in histone demethylase mutants. To the left metaplot for H3K27me3 levels for genes both up-regulated and hypo-methylated and to the right metaplot of H3K27me3 levels in genes both down-regulated and hyper-methylated. Top panel, *ref6-5*; Bottom panel, *elf6-C*. p-values for Fisher’s exact test are shown in brackets, N.S. Not Significant.

The deposition of H3K27me3 by PRCs correlates with transcriptional repression in plants and animals. The dynamic regulation of this epigenetic mark enables the reactivation of genes primarily implicated in developmental programs; thus, any disruption to these regulatory networks results in major developmental aberrations (Kassis et al., 2017; Lewis, 1978; Molitor et al., 2016). The demethylation of H3K27me3 has been linked to the enzymatic activity of five JMJ-type proteins, which act antagonistically to SET-domain histone methyltransferases from the PRC2 complex (Yan et al., 2018). To gain further knowledge about these processes in *Arabidopsis*, we investigated the function of two sequence-related histone demethylases, ELF6 and REF6. To aid this analysis, we isolated a loss-of-function T-DNA insertion of *REF6* (*ref6-5*) and a targeted CRISPR/Cas9 deletion in the first exon of *ELF6* (*elf6-C*) (See Materials and methods and Figure 1—figure supplement 1). Similar to previous reports, we found that our *elf6-C* plants displayed an early flowering phenotype characterized by a reduced number of rosette leaves at bolting (Jeong et al., 2009; Noh et al., 2004). Conversely, *ref6-5* plants displayed a late flowering phenotype and an increased number of rosette leaves at bolting stage (Figure 1A and Figure 1—figure supplement 2A–B). By manual crossing, we generated *elf6-C/ref6-5* double mutant plants, which displayed pleiotropic growth phenotypes, including increased number of petals and pleiotropic defects in leaf morphology, such as serrations and downward curling (Figure 1A and Figure 1—figure supplement 2C–D). These phenotypes were akin to those recently reported for independently generated double mutants of the same histone demethylases (Yan et al., 2018), and their stability was confirmed by generating double mutants using different mutant allele combinations (Figure 1—figure supplement 2E). In all combinations tested, double mutant plants displayed a reduction in silique length (Figure 1B and Figure 1—figure supplement 2E), thus suggesting that these mutations redundantly affect plant fertility. Microscopy analysis of developing seeds revealed that while embryo development in *elf6-C* was normal, seeds from *ref6-5* and *elf6-C/ref6-5* contained embryos with patterning defects (Figure 1B). However, these embryonic abnormalities did not affect seed germination rates (Figure 1—figure supplement 2F).

**Figure 1—figure supplement 1.**
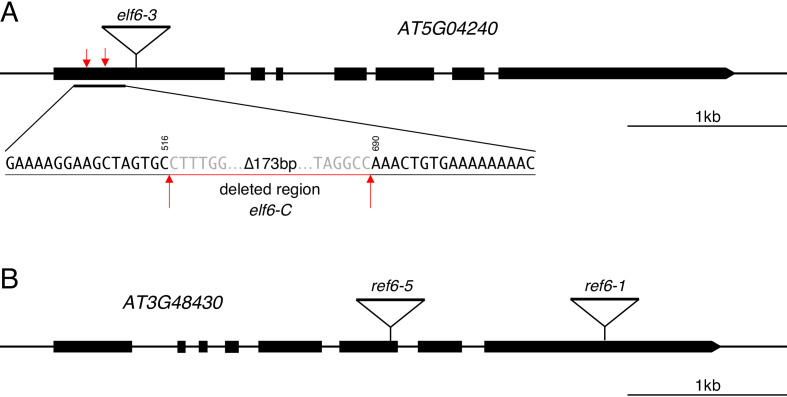
Graphical representation of mutant alleles for *ELF6* and *REF6* used in this study. (**A**) Schematic diagram of *ELF6* locus showing the location of different mutant alleles: *elf6*-3 is a T-DNA insertion (SALK_074694) in the first exon and *elf6-C* is a CRISPR/Cas9 deletion in the first exon that leads to an early stop codon. Black boxes represent exons, triangle shows the insertion site for *elf6*-3 and red arrows mark the start and end of the deleted region in *elf6-C*. Scale bar, 1 kb. (**B**) Schematic diagram of *REF6* locus showing the location of different mutant alleles: *ref6-5* (GABI_70E03) and *ref6*-1 (SALK_001018) are T-DNA insertions in the sixth and last exon, respectively. Black boxes represent exons and triangles shows the T-DNA insertion sites. Scale bar, 1 kb.

**Figure 1—figure supplement 2.**
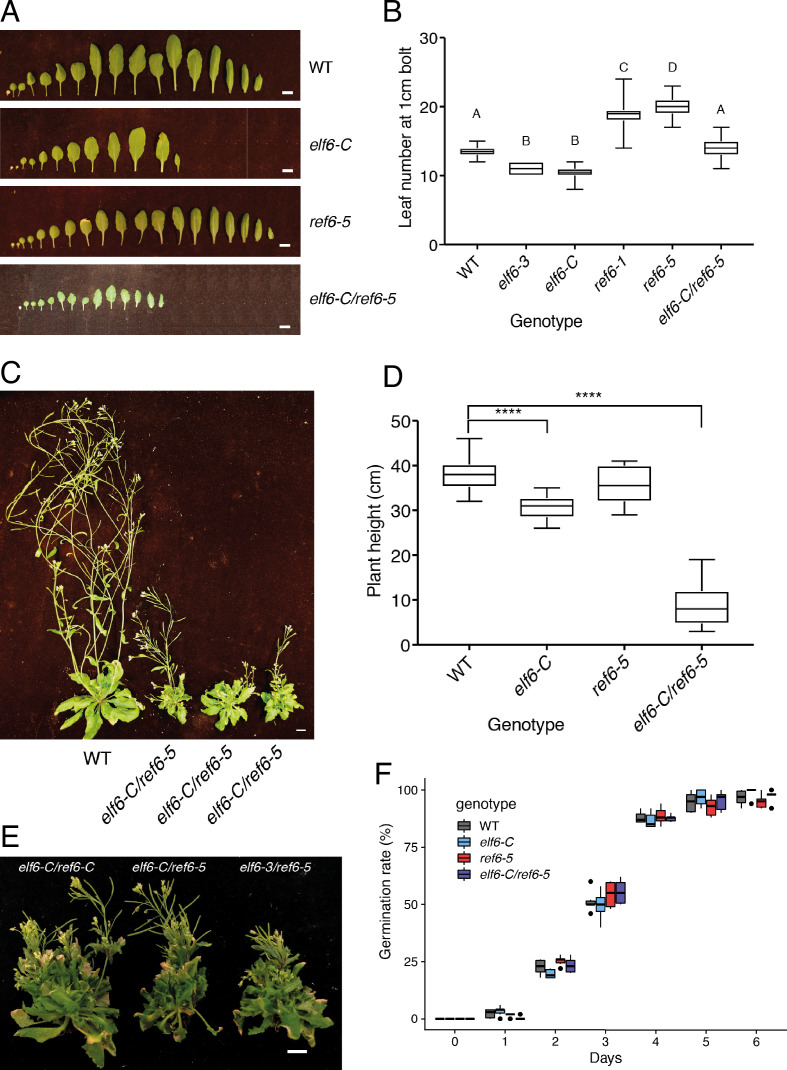
Phenotypic characterization of *elf6-C*, *ref6-5* and double mutants. (**A**) Rosette leaves at bolting stage of Arabidopsis wild-type (WT) and histone demethylase mutants (*elf6-C*, *ref6-5* and *elf6-C*/*ref6-5*). Scale bars, 1 cm. (**B**) Boxplot for leaf number at bolting stage for wild-type (WT) and different mutants. Letters represent groups of statistically significantly different samples. Differences between genotypes determined by Students *t*-test, using a sample size of n = 30. (**C**) Growth phenotypes of wild-type (WT) and *elf6-C*/*ref6-5* mutant. Scale bar, 1 cm. (**D**) Boxplot of plant height for wild-type (WT) and histone demethylase mutants. Asterisks represent significant differences between samples. Differences between genotypes determined by Students *t*-test test, ****p<0.001, sample size of n = 30. (**E**) Growth phenotypes of *elf6-C*/*ref6-C*, *elf6-C*/*ref6-6* and *elf6-3*/*ref6-5* double mutants. Scale bar, 1 cm. (**F**) Boxplot of seed germination rates in wild-type (WT) and histone demethylase mutants. Germination was scored as radicle protrusion through seed coat. n = 300 from six biological replicates.

**Figure 1—figure supplement 3.**
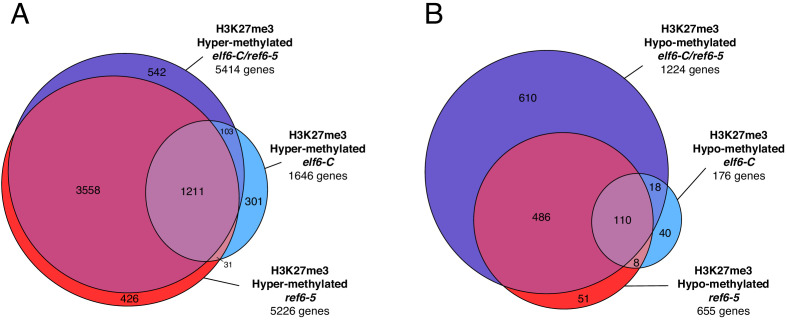
Genes with differential K3K27me3 methylation in histone demethylase mutants. (**A**) Euler diagram of H3K27me3 hyper-methylated genes in histone demethylase mutants compared to wild-type (WT). (**B**) Euler diagram of H3K27me3 hypo-methylated genes in histone demethylase mutants compared to wild-type (WT).

**Figure 1—figure supplement 4.**
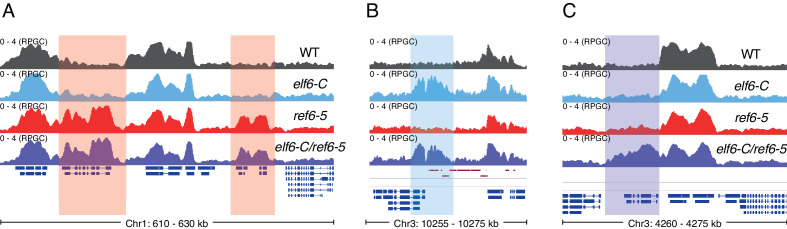
Genomic regions accumulating K3K27me3 in histone demethylase mutants. (**A**) Genome browser view of ChIP-seq signal as normalized reads per genomic content (RPGC). Shaded red boxes, genes targeted by REF6. (**B**) Genome browser view of ChIP-seq signal as normalized reads per genomic content (RPGC). Shaded blue box, gene targeted exclusively by ELF6. (**C**) Genome browser view of ChIP-seq signal as normalized reads per genomic content (RPGC). Shaded purple boxes, genes targeted by both REF6 and ELF6, and only hyper-methylated in the double mutant *elf6-C*/*ref6-5*.

**Figure 1—figure supplement 5.**
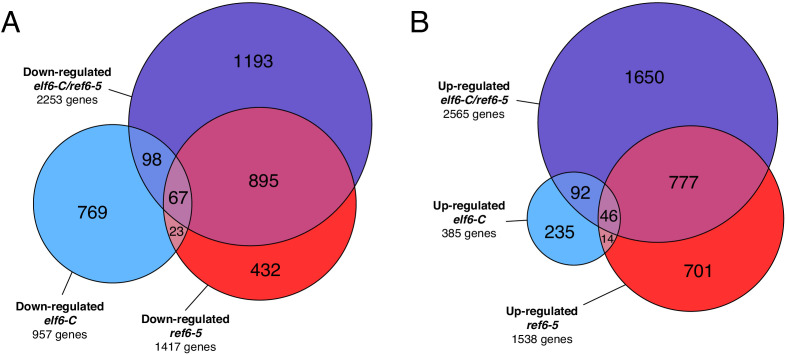
Genes differentially expressed in histone demethylase mutants. (**A**) Euler diagram of down-regulated genes in histone demethylase mutants compared to wild-type (WT). (**B**) Euler diagram of up-regulated genes in histone demethylase mutants compared to wild-type (WT).

**Figure 1—figure supplement 6.**
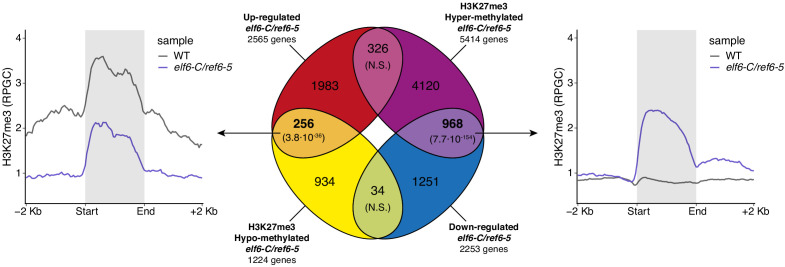
Relation between hypermethylation of H3K27me3 and downregulation of genes in *elf6-C*/*ref6-5*. Venn diagram showing overlap between differentially expressed genes (DEGs) and H3K27me3 differentially methylated genes in *elf6-C*/*ref6-5*. Venn diagram showing overlap between differentially expressed genes (DEGs) and H3K27me3 differentially methylated genes in *elf6-C*/*ref6-5*. To the left metaplot for H3K27me3 levels for genes both up-regulated and hypo-methylated and to the right metaplot of H3K27me3 levels in genes both down-regulated and hyper-methylated. p-values for Fisher’s exact test are shown in brackets, N.S. Not Significant.

REF6 is thought to act as a H3K27me3 demethylase and a positive regulator of gene expression (Hou et al., 2014; Li et al., 2016; Lu et al., 2011; Wang et al., 2019), while the role of ELF6 remains poorly understood. To shed light on the function of these two proteins, we analyzed the distribution of H3K27me3 in *elf6-C, ref6-5* and *elf6-C/ref6-5* seedlings through a ChIP-seq assays and compared them to that in wild-type plants. Overall, the accumulation of H3K27me3 within genes was more pronounced in *ref6-5* than in *elf6-C* (Figure 1C). Most of the hyper-methylated genes found in *elf6* (75%) were hyper-methylated to a greater extent in both *ref6-5* and *elf6-C/ref6-5*, suggesting that these histone demethylases have partially overlapping yet distinct roles in the control of H3K27me3 homeostasis in Arabidopsis (Figure 1D and Figure 1—figure supplements 3–4). In order to further understand the role of ELF6 and REF6 in transcriptional regulation, we performed an RNA-seq analysis. When combining transcriptomic and H3K27me3 ChIP-seq data, we found a strong correlation primarily between genes that were both hyper-methylated at H3K27me3 and down-regulated, thus indicating that this epigenetic mark contributes to their transcriptional repression (Figure 1E and Figure 1—figure supplements 5–6). We also found genes that were hypo-methylated and up-regulated, which could be linked to the global transcriptional deregulation observed in these mutants. Taken together, our data point to the essential, yet distinct, roles of REF6 and ELF6 in H3K27me3 homeostasis at genic regions of the Arabidopsis genome.

### REF6 controls H3K27me1 homeostasis in euchromatin

**Figure 2.**
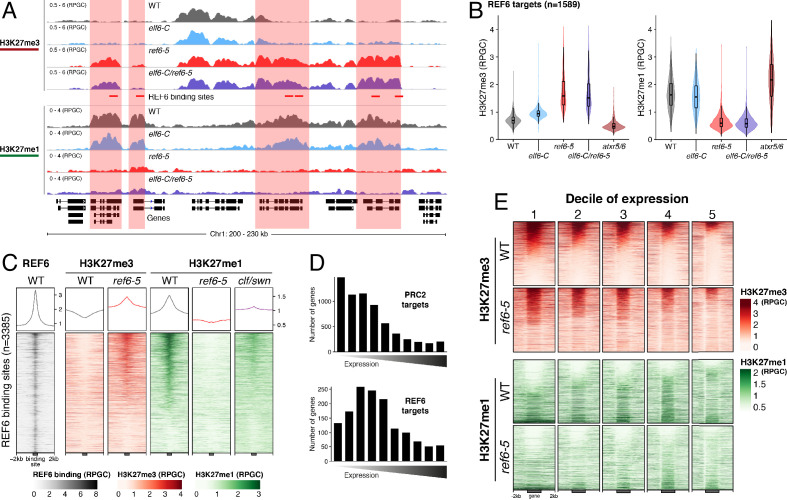
Arabidopsis REF6 plays an essential role in the deposition of H3K27me1 in active chromatin. (**A**) Genome browser views of background subtracted ChIP-seq signals for H3K27me3 and H3K27me1 as normalized reads per genomic content (RPGC) in wild-type (WT) and histone demethylase mutants (*elf6-C*, *ref6-5* and *elf6-C*/*ref6-5*). Shaded boxes, genes targeted exclusively by REF6. (**B**) Violin plots showing the distribution of H3K27me3 and H3K27me1 on genes targeted by REF6. Genes were categorised as targeted if a H3K27me3 peak was annotated on them in *ref6-5* and in *elf6-C*/*ref6-5* but not in WT. (**C**) Heatmap showing the distribution of H3K27me3 and H3K27me1 on genomic sequences targeted by REF6 for wild-type (WT), *ref6-5*, and *clf/swn* plants. Sample size n = 3385. (**D**) Bar charts showing the number of genes for different expression quantiles predicted to be targeted by PRC2 and REF6. (**E**) Heatmap showing the distribution of H3K27me3 and H3K27me1 present on genes corresponding to low-expression (1-5) quantiles. .

Biochemical analyses have revealed that REF6 can remove both tri- and di-methyl groups but not mono-methyl groups at lysine 27 on histone 3 (Lu et al., 2011). We therefore hypothesized that, in addition to controlling H3K27me3 homeostasis, REF6 and ELF6 may be also implicated in H3K27me1 homeostasis. To test this hypothesis, we determined the distribution of H3K27me1 through ChIP-seq assays and found that most of the genes targeted by REF6 accumulate high levels of H3K27me1 in wild-type (Figure 2A–C and Figure 2—figure supplement 1). Because the deposition of H3K27me1 in Arabidopsis is thought to be mediated by ATXR5 and 6 (Jacob et al., 2009; Jacob et al., 2010), we determined the genomic distribution of H3K27me1 in the hypomorphic *atxr5/atxr6* mutant. As previously described, large-scale H3K27me1 accumulation in these mutants was significantly reduced at pericentromeric heterochromatin (Figure 2—figure supplement 2). However, we noticed that in *atxr5/atxr6* lines the levels of this histone mark increased in gene-rich regions, pointing to the existence of an alternative pathway for H3K27me1 deposition in euchromatin. These data led us to postulate that the maintenance of H3K27me1 at euchromatin could be mediated by REF6. We therefore investigated the relationship between H3K27me1 and H3K27me3 at genes targeted by REF6. We found that the loss of REF6 activity results in both the accumulation of H3K27me3 and a drastic reduction in H3K27me1 at those loci, while the loss of ELF6 did not have an effect (Figure 2B). We next assessed genomic regions directly targeted by REF6 (Cui et al., 2016; Li et al., 2016) and found that the accumulation of H3K27me3 in *ref6-5* was associated with a complete loss of H3K27me1 (Figure 2C). Taken together these data revealed that the maintenance of H3K27me1 in euchromatin is dependent on REF6. To test if the H3K27me1 present in REF6 binding sites is produced by the sequential methylation by PRC2 and partial demethylation by REF6, we performed ChIP-seq analyses for H3K27me1 in the PRC2 methyltransferase double mutant *clf/swn.* We found a strong reduction of H3K27me1 in these mutants at REF6 binding sites (Figure 2C), thus confirming that H3K27me1 at these euchromatic sites is PRC2-dependant.

**Figure 2—figure supplement 1.**
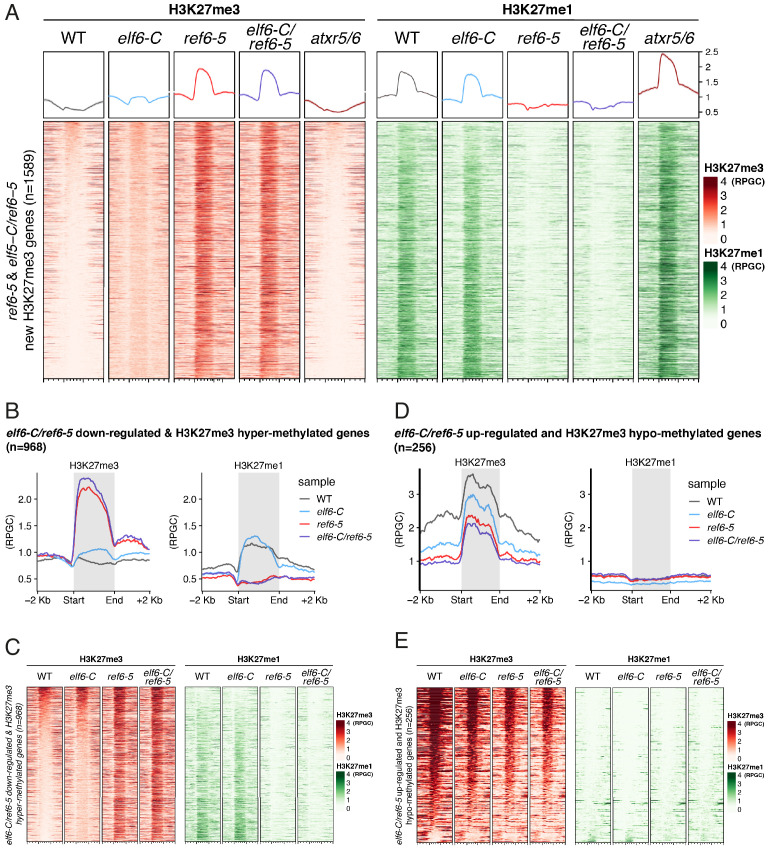
REF6 catalyses H3K27me3 to H3K27me1 conversion in genes causing de-repression. (**A**) Heatmap showing the distribution of H3K27me3 (red) and H3K27me1 (green) on genes targeted by REF6. Genes were categorised as targets if a H3K27me3 peak was annotated on them in *ref6-5* and in *elf6-C*/*ref6-5* but not in WT. n = 1589. Intensity of the colour represents RPGC of ChIP-seq. Genes are sorted by the amount of H3K27me3 in WT. Boxes at the top represent metaplots of the average signal for all these genes. (**B**) Metaplot of median of H3K27me3 and H3K27me1 across both downregulated and hypermethylated genes in *elf6-C*/*ref6-5* for wild-type (WT) and histone demethylase mutants, n = 968. (**C**) Heatmap of the same genes as Fig S 7B. Intensity of the colour represents ChIP-seq signal as RPGC. (**D**) Metaplot of median of H3K27me3 and H3K27me1 across both upregulated and hypomethylated genes in *elf6-C*/*ref6-5* for wild-type (WT) and histone demethylase mutants, n = 256. (**E**) Heatmap of the same genes as Fig S 7D. Intensity of the colour represents ChIP-seq signal as RPGC.

**Figure 2—figure supplement 2.**
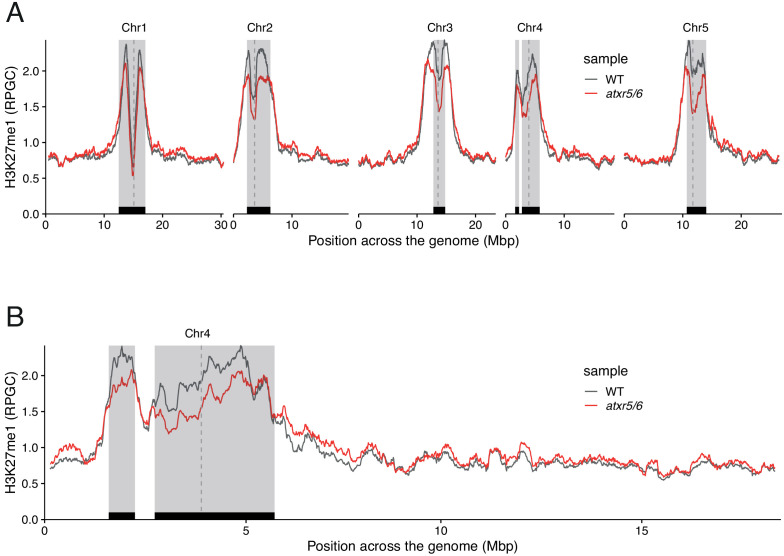
ATXR5/6 contributes to the deposition of H3K27me1 in pericentromeric regions. (**A**) Distribution of H3K27me1 across chromosomes of wild-type (WT) and *atxr5/6* mutants. Grey shaded boxes, pericentromeric regions. (**B**) Distribution of H3K27me1 across Chromosome 4 of Arabidopsis genome in wild-type (WT) and *atxr5/6* mutants. Grey shaded boxes, pericentromeric regions.

**Figure 2—figure supplement 3.**
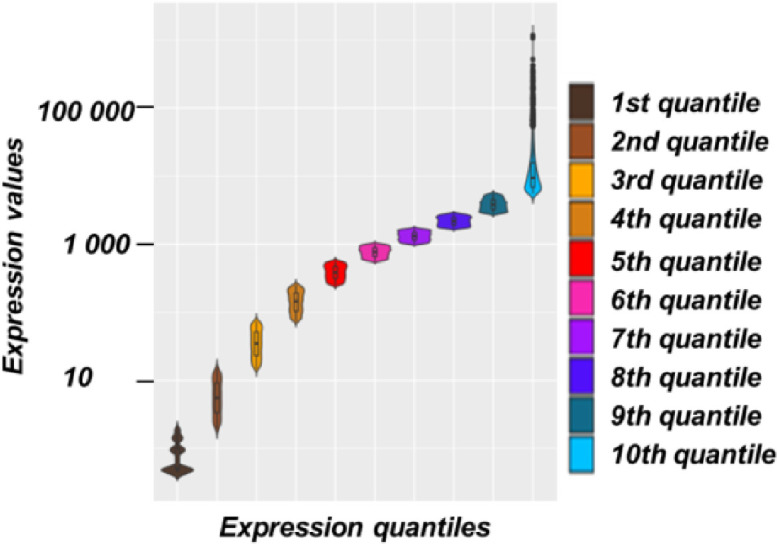
Division of Arabidopsis genes according to their levels of expression. Violin plot with boxplots representing the expression levels of genes in Arabidopsis divided in 10 quantiles.

**Figure 2—figure supplement 4.**
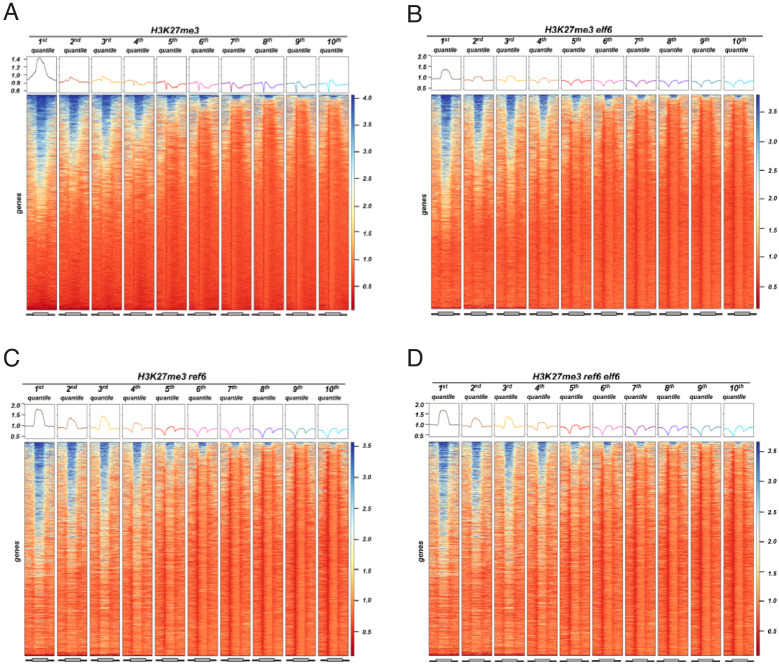
Histone demethylase mutants show an increase in H3K27me3 on genes with intermediate levels of expression Heatmaps showing the H3K27me3. ChIP-seq signal across genes split by 10 quantiles of expression for wild-type (WT) and histone demethylase mutants. Boxes on top represent metaplots of the median signal in each quantile. Genes are sorted by the amount of H3K27me3 in WT. (**A**) WT. (**B**) *elf6-C*. (**C**) *ref6-5*. (**D**) *elf6-C*/*ref6-5*.

**Figure 2—figure supplement 5.**
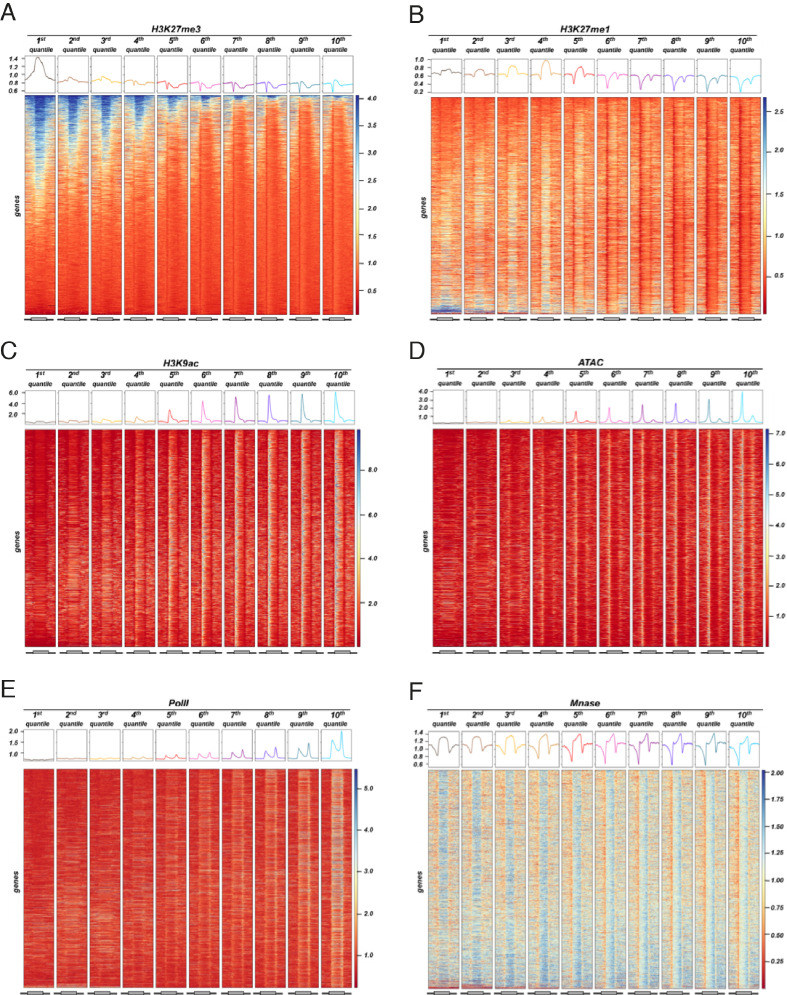
Distribution of different epigenetic features for different expression quantiles. Heatmaps showing signal for different epigenetic features across 10 expression quantiles for wild-type. Boxes on top represent metaplots of the median signal in each quantile. Genes in all panels are always sorted by the amount of H3K27me3. (**A**) H3K27me3. (**B**) H3K27me1. (**C**) H3K9ac. (**D**) ATAC-seq. (**E**) PolII-seq. (**F**) Mnase-seq.

Although it is well known that H3K27me1 in Arabidopsis contributes to the repression of heterochromatic TEs, its role in euchromatin remains unknown. To address this gap in our knowledge, we examined the relationship between REF6-dependent H3K27me1 deposition and transcription. To aid this analysis, we divided the transcriptome into 10 equal deciles according to their transcriptional state (Figure 2—figure supplement 3). We found that while H3K27me3 was primarily associated with strongly repressed genes in wild-type (first three quantiles), in *ref6-5*, the ectopic accumulation of H3K27me3 primarily affected genes that displayed low levels of expression (second to fifth quantiles) (Figure 2D and Figure 2—figure supplement 4). Moreover, we found that the activity of REF6 was required for low-level expression genes (third to fifth quantiles) (Figure 2E and Figure 2—figure supplement 5). Collectively, these data support the view that REF6 contributes to gene activation, by the removal of PRC2-dependent repressive marks, and to the maintenance of low-level basal expression, by maintaining H3K27me1 in transcriptionally active chromatin.

### Inheritance of ectopic H3K27me3 imprints alters the epigenome

**Figure 3.**
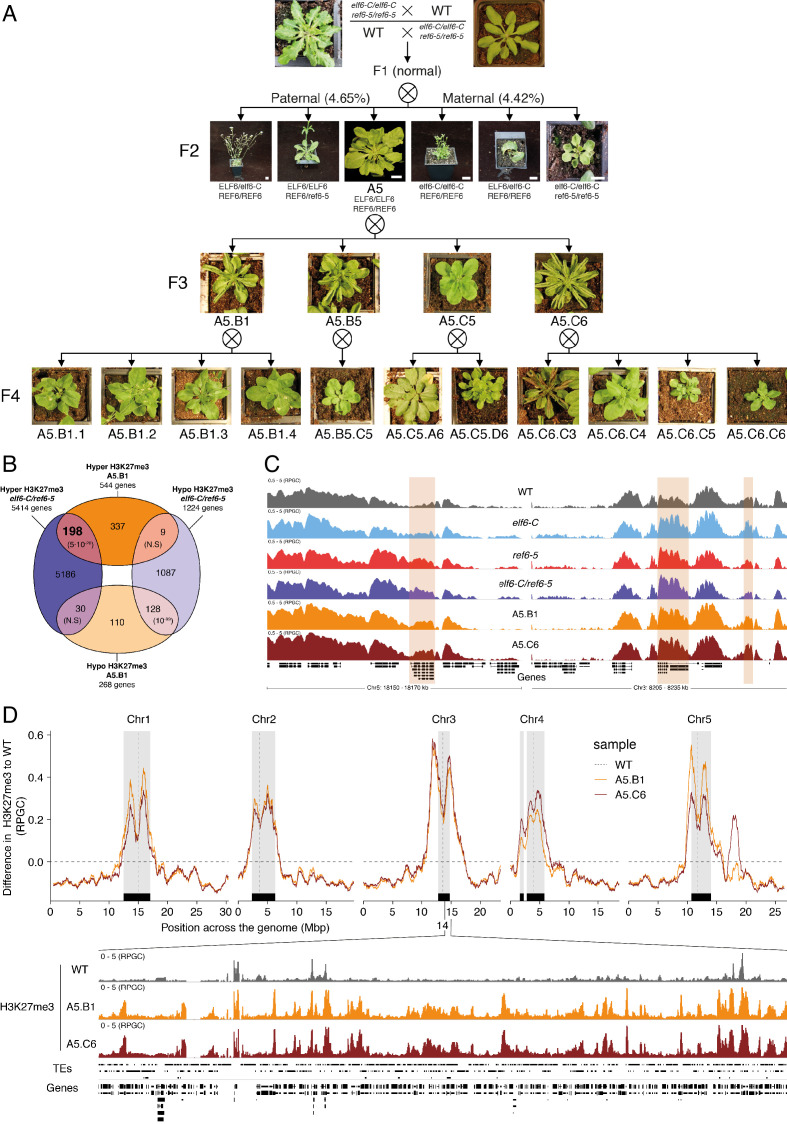
Pleiotropic developmental abnormalities associated with the inheritance of ectopic H3K27me3 imprints in Arabidopsis. (**A**) The F_2_ hybrids from reciprocal crosses between wild-type (WT) and *elf6-C/ref6-5* display novel abnormal plant growth phenotypes. Frequency of abnormal phenotypes according to parental transmission of mutant alleles is indicated. Pedigree of an epimutant that was genetically wild-type for ELF6 and REF6 and selected for genomic analysis after propagation by selfing. Scale bars, 1 cm. (**B**) Venn diagram showing the overlap in genes accumulating H3K27me3 in *elf6-C/ref6-5* and F_5_ progenies from A5.B1. p-values for Fisher’s exact test are shown in brackets, N.S. Not Significant. (**C**) Genome browser views of background subtracted ChIP-seq signals for H3K27me3 as normalized reads per genomic content (RPGC) in wild-type (WT), *elf6-C*, *ref6-5*, *elf6-C/ref6-5*, and F_5_ progenies from A5.B1 and A5.C6. Shaded boxes, genes showing transgenerational inheritance of H3K27me3. (**D**) Top panel: Differences in the chromosomal distribution of H3K27me3 as normalized reads per genomic content (RPGC) between F5 progenies from A5.B1 and A5.C6 and wild-type (WT). Grey shaded boxes, pericentromeric regions. Bottom panel: Genome browser view of ChIP-seq signal for H3K27me3 as normalized reads per genomic content (RPGC) in wild-type (WT), and F_5_ progenies from A5.B1 and A5.C6 in a pericentromeric region.

It has been shown in Arabidopsis that histone demethylases are critical for the resetting of H3K27me3 across generations (Crevillén et al., 2014; Gan et al., 2014; Liu et al., 2019; Zheng et al., 2019). To understand the biological significance of this epigenetic resetting thought to take place during plant sexual reproduction, we generated reciprocal crosses between single and double histone demethylase mutants and wild-type plants. We found that F_1_ plants from these crosses were indistinguishable from wild-type, however some F_2_ plants displayed unexpected developmental phenotypes that were not present in either single or double mutants (4.65% paternal and 4.42% maternal transmission, n = 1500 each) (Figure 3A). On the other hand, the frequency of plants displaying developmental abnormalities in the F_2,_ resulting from F_1_ hybrids between wild-type and single histone demethylase mutants, was markedly lower (0.58% for *elf6-C* and 0.63% for *ref6-5;* n > 300 each). Intriguingly, abnormal plants arising from the *elf6-C/+;ref6-5/+* hybrids segregated for the different mutant allele combinations (Figure 3A), thus indicating that these novel phenotypes are not genetically linked to either *elf6-C* or *ref6-5* mutations. These plants displayed an array of developmental abnormalities, including abnormal leaf and inflorescence development and a severe reduction in fertility. We therefore reasoned that these defects could be caused by epimutations arising in *elf6-C/ref6-5* due to defects in H3K27me3 resetting during sexual reproduction (Figure 3A). In support of this hypothesis, we identified among these F_2_ progenies one line (A5) that was genetically wild-type for ELF6 and REF6, but had enlarged rosette leaves and partial fertility. From this line, the viable seeds were used to propagate single-seed descent progenies by self-pollinating for over two generations. Plants from F_3_ progenies displayed a wide spectrum of developmental abnormalities ranging from plants with wild-type characteristics (21.7%, n > 100 each) to partially infertile plants with abnormal rosette leaves (78.3%, n > 100 each). Notably, F_4_ and F_5_ progenies continued to segregate the broad range of developmental phenotypes (Figure 3—figure supplement 1). These data indicate that loss of histone demethylase activity causes the accumulation of epimutations. To test this working hypothesis, we grew seedlings from two different F_5_ progenies (A5.B1 and A5.C6) and performed a ChIP-seq analysis to determine H3K27me3 distribution across the genome. This analysis revealed 544 euchromatic loci with elevated levels of H3K27me3, of which one third were also found to be hyper-methylated in the parental double mutant line used in reciprocal crosses (Figure 3B). These data suggest that some of the H3K27me3 imprints present in epimutants were established in *elf6-C/ref6-5* and were then stably transmitted over the five generations, even after the wild-type function of these histone demethylases had been restored (Figure 3C and Figure 3—figure supplement 2). We herein named these lines *epiERs* (epimutants arising from *elf6-C/ref6-5)*. Notably, for both characterized lines we found that the ectopic accumulation of H3K27me3 was particularly common and strong in constitutive heterochromatin within the pericentromeric regions (Figure 3D). Taken together, our data suggest that ELF6 and REF6 are necessary to limit the transmission of H3K27me3 imprints to offspring, and that failure to do so results in epigenomic and developmental abnormalities.

**Figure 3—figure supplement 1.**
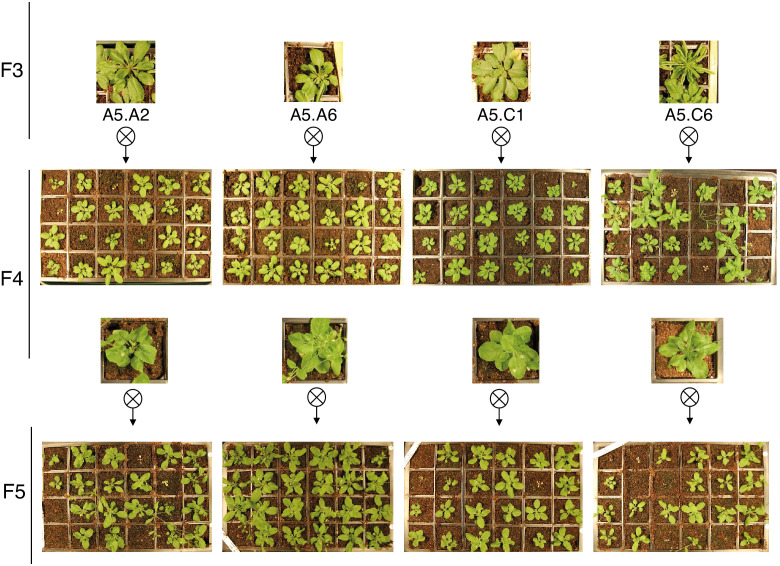
Phenotypic variation present in epiER progenies. Representative images of F_4_ and F_5_ progenies from epiER line A5 showing a wide range of developmental phenotypes.

**Figure 3—figure supplement 2.**
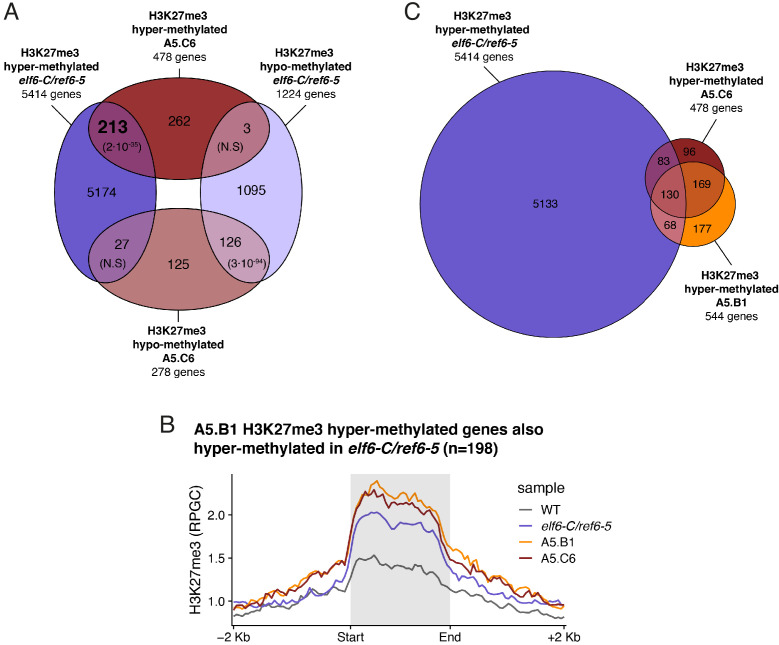
Inheritance of ectopic H3K27me3 in *epiERs*. (**A**) Venn diagram showing the intersection between genes showing accumulation of H3K27me3 in *elf6-C*/*ref6-5* and *epiER* A5.C6. p-values for Fisher’s exact test are shown in brackets, N.S. Not Significant. (**B**) Metaplot of the median of ChIP-seq RPGC across genes hypermethylated in both *elf6-C*/*ref6-5* and *epiER* A5.C6. n = 198. (**C**) Euler diagram showing the intersection between the genes hypermethylated in *elf6-C*/*ref6-5* and *epiERs* A5.B1 and A5.C6.

### Accumulation of ectopic H3K27me3 at centromeric heterochromatin is linked to DNA hypomethylation in epiERs

**Figure 4.**
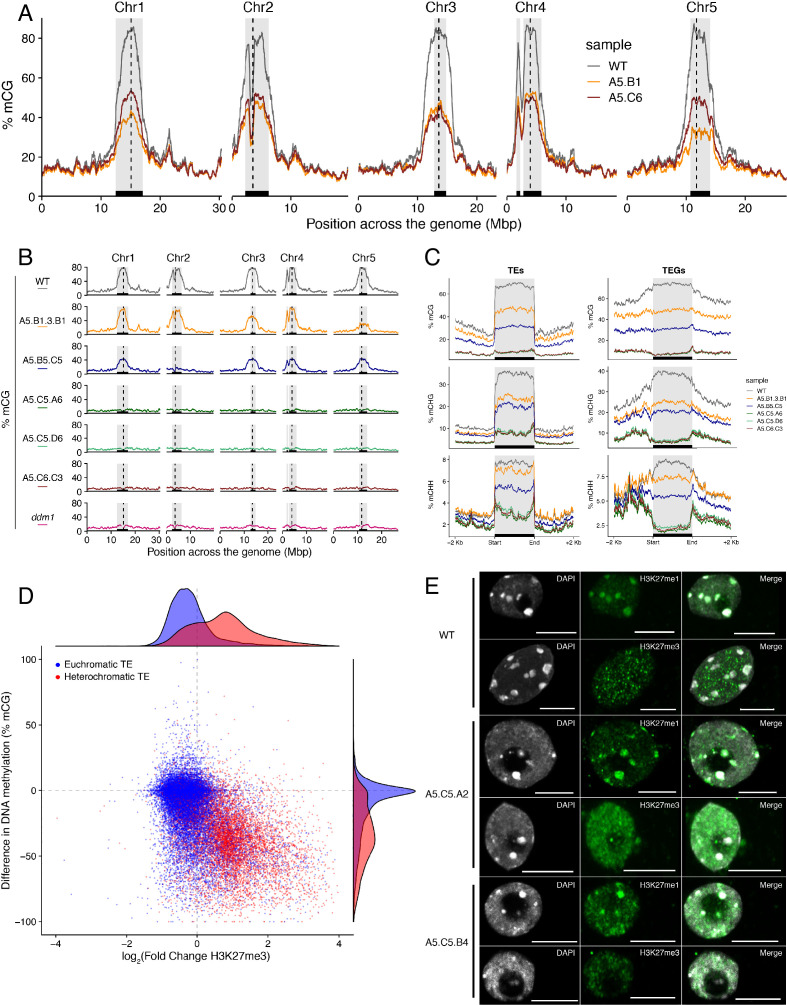
Ectopic accumulation of H3K27me3 is associated with the loss of DNA methylation at pericentromeric heterochromatin and affects chromatin condensation. (**A**) Distribution of DNA methylation across chromosomes of wild-type (WT) and progenies from *epiER*s A5.B1 and A5.C6. Grey shaded boxes, pericentromeric regions. (**B**) Distribution of DNA methylation across chromosomes of individual plants from wild-type (WT), *ddm1*, and *epiER*s A5.B1.3.B1, A5.B5.C5, A5.C5.A6, A5.C5.D6 and A5.C6.C3. Grey shaded boxes, pericentromeric regions. (**C**) Distribution of DNA methylation across Transposable Elements (TEs) and Transposable Element Genes (TEGs) of individual plants from wild-type (WT) and *epiER*s A5.B1.3.B1, A5.B5.C5, A5.C5.A6, A5.C5.D6 and A5.C6.C3. Black box, centromeric regions. (**D**) Correlation between DNA methylation changes and H3K27me3 changes on euchromatic and heterochromatic TEs, in wild-type (WT) and *epiER* A5.B1. (**E**) Immunolocalization showing the distribution of H3K27me3 and H3K27me1 in interphase nuclei of wild-type, A5.C5.A2 and A5.C5.B4 plants. Scale bars, 5 μm.

Loss of DNA methylation has been linked to the abnormal deposition of H3K27me3 in heterochromatin (Batista and Köhler, 2020; Mathieu et al., 2005). However, mutants defective in H3K27me3 deposition do not display altered global DNA methylation levels (Stroud et al., 2014). To test if the ectopic accumulation of H3K27me3 found in *epiER*s could affect DNA methylation, we performed a BS-seq analysis on the two F_5_ epimutant progenies used for the ChIP-seq analysis. We found that both *epiER* lines displayed global reductions in DNA methylation, primarily at pericentromeric regions (Figure 4A). This global reduction in methylation occurred despite there being no ectopic accumulation of H3K27me3 at any genes involved in the DNA methylation pathway, including *MET1*, *DDM1*, *CMT2* and *CMT3*, in the parental mutant. In addition, we found that the two analysed *epiERs* displayed notable differences in DNA methylation levels between lines and among chromosomes (Figure 4A).

**Figure 4—figure supplement 1.**
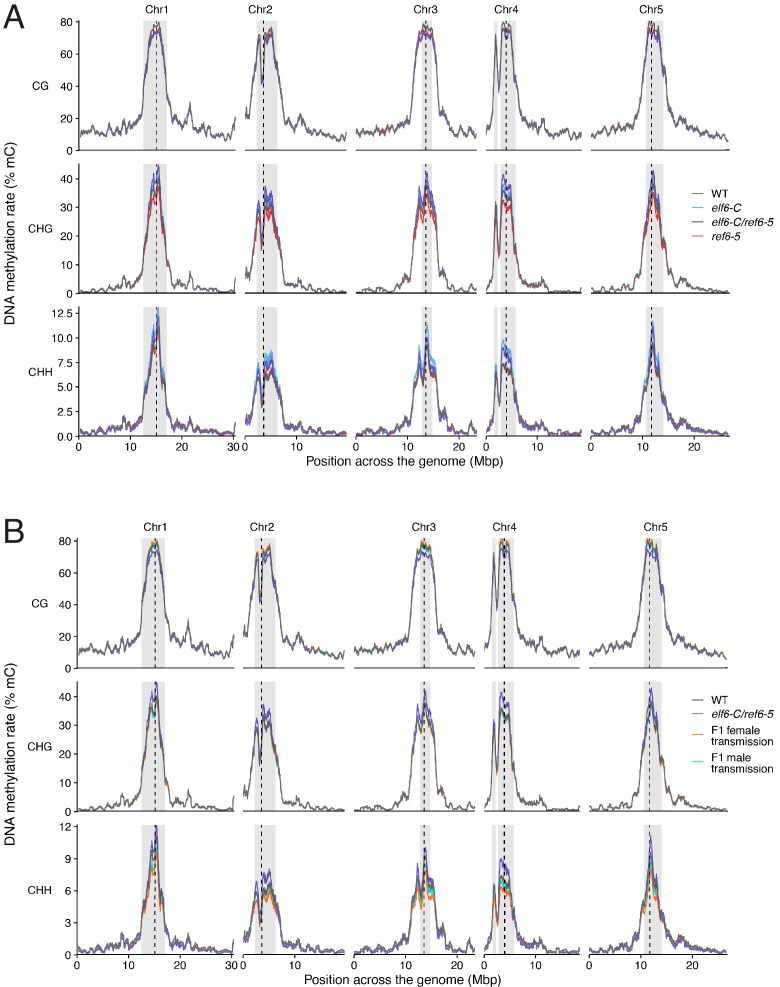
Chromosomal distribution of DNA methylation in histone demethylase mutants and F1 hybrids. (**A**) DNA methylation in wild-type (WT), *elf6-C*, *ref6-5* and *elf6-C*/*ref6-5* plants. (**B**) DNA methylation in wild-type (WT) and F1 hybrids from reciprocal crosses between wild-type and *elf6-C*/*ref6-5*. Paternal or maternal transmission of mutant alleles is indicated.

**Figure 4—figure supplement 2.**
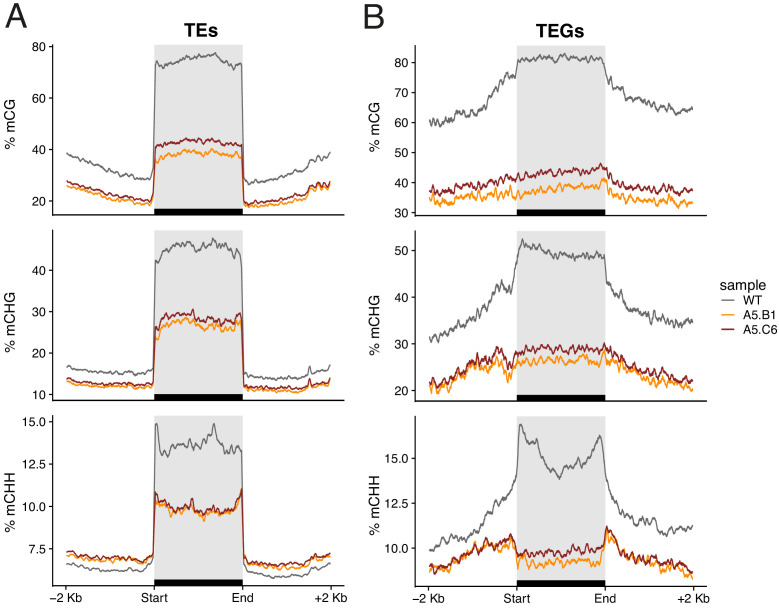
DNA hypomethylation of transposable elements in *epiERs*. Metaplot showing the proportion of DNA methylation across transposable elements (**A**) and transposable element genes (**B**) for wild-type (WT) and progenies from two *epiERs*.

**Figure 4—figure supplement 3.**
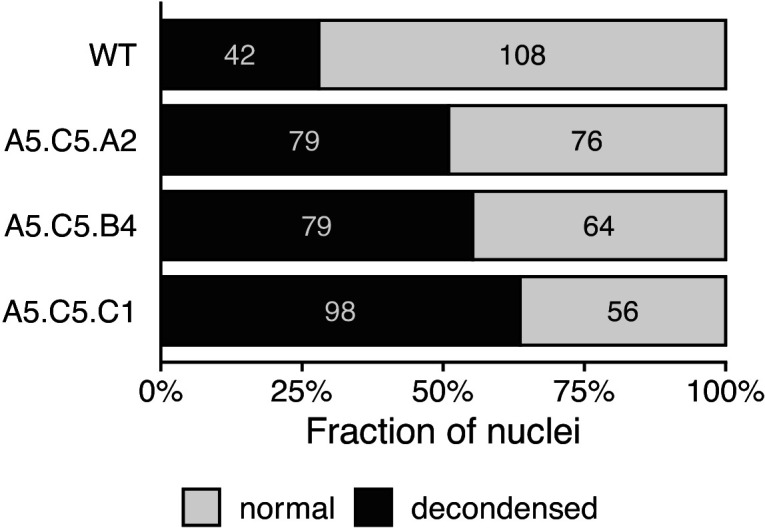
Condensation of chromatin in *epiERs*. Bar plot showing the fraction of nuclei categorised as decondensed after DAPI staining in wild-type (WT) and *epiERs* A5.C5.A2, A5.C5.B4 and A5.C5.C1. Number of nuclei for each category in each line shown inside plot.

To test whether the reduction in DNA methylation found in *epiERs* could arise from defects already present in histone demethylation mutants, we analyzed the DNA methylation levels in the genomes of *elf6-C, ref6-5* and *elf6-C/ref6-5* mutants. Importantly, we did not find any significant changes in DNA methylation in any of these mutants (Figure 4—figure supplement 1A). We therefore investigated variation in DNA methylation between plants within each population by performing a BS-seq analysis on individual F_4_ *epiER* plants. This analysis revealed that while some plants were consistently devoid of DNA methylation at pericentromeric regions, similar to the *ddm1* mutant, others displayed intermediate states that varied from chromosome to chromosome, thus providing evidence for a partial resetting of DNA methylation (Figure 4B). Notably, the loss of DNA methylation in constitutive heterochromatic regions in *epiERs* was associated with a decrease in methylation at TEs and genes located therein (Figure 4C and Figure 4—figure supplement 2). Moreover, these defects in heterochromatin methylation were also observed in F_1_ hybrids derived from reciprocal crosses between wild-type and histone demethylase mutants (Figure 4—figure supplement 1B). Given that these pericentromeric regions in *epiERs* failed to fully recover DNA methylation to wild-type levels and displayed elevated H3K27me3 levels, we hypothesized that they may be partially protected from the DNA methylation activity commonly used to target transposons and repetitive DNA elements in plants (Matzke and Mosher, 2014). We therefore investigated the relationship between DNA methylation and H3K27me3 on transposons located in euchromatic and constitutive heterochromatic genome regions. We found that in *epiERs*, heterochromatic TEs that gained H3K27me3 had a proportional loss of DNA methylation whereas euchromatic TEs showed no change in DNA methylation (Figure 4D). These data support the view that a gain in H3K27me3 has a negative effect on the deposition and/or the maintenance of DNA methylation at heterochromatic transposons. To evaluate the extent to which these defects may affect chromatin compaction, we performed immunostaining assays on interphase nuclei using specific antibodies. We found that in *epiERs,* heterochomatin compaction is strongly affected and manifest as a higher proportion of decondensed nuclei compared to wild type (Figure 4E; Figure 4—figure supplement 3). Collectively, these data suggest that the ectopic accumulation of H3K27me3 in *epiERs* results in pericentromeric heterochromatin defects.

### Epigenomic defects result in transcriptional activation of pericentromeric loci and genome instability

**Figure 5.**
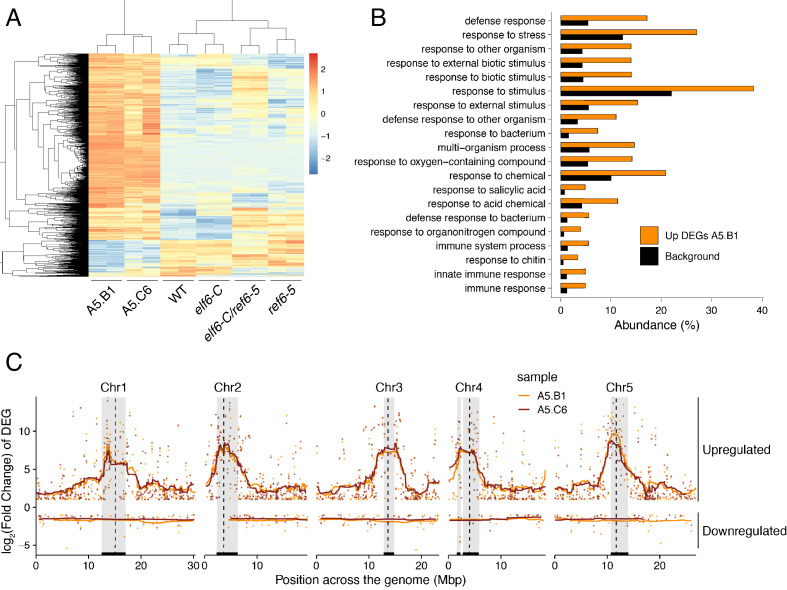
Global upregulation of centromeric gene expression in *epiER*s. (**A**) Heatmap showing scaled expression levels of Differentially Expressed Genes between wild-type and progeny of epi*ER* A5.B1 in wild-type (WT) *elf6-C*, *ref6-5*, *elf6-C/ref6-5*, and progenies of *epiER*s A5.B1 and A5.C6. (**B**) Gene Ontology analysis showing the functional categories enriched in genes upregulated in progeny of *epiER*s A5.B1. (**C**) Differential gene expression across each Arabidopsis chromosome for genes upregulated and downregulated in progenies of *epiER*s A5.B1 and A5.C6. Grey shaded boxes, pericentromeric regions.

**Figure 6.**
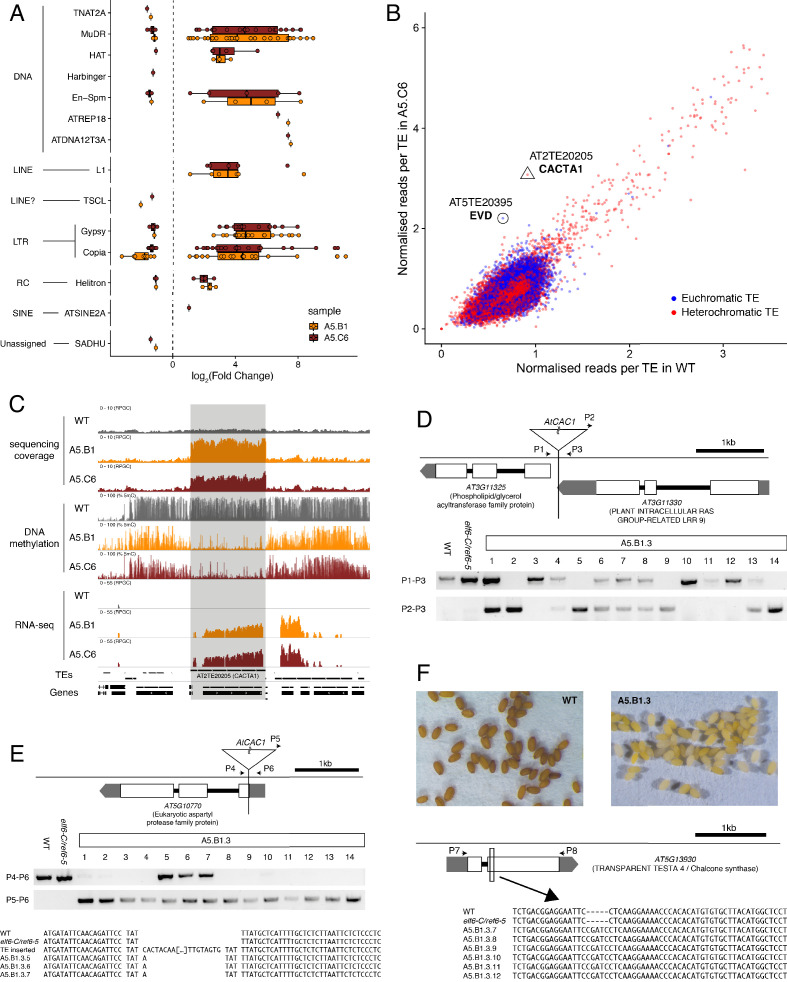
Transposon mobilization in *epiER*s results in heritable genetic lesions. (**A**) Differential expression of DNA and RNA transposon families grouped by superfamily in progenies of *epiER*s A5.B1 and A5.C6. (**B**) Copy number variation of transposons in progenies of *epiER* A5.C6. Blue dots, euchromatic TEs; Red dots, heterochromatic TEs. (**C**) Genome browser views of normalized sequencing coverage (RPGC), DNA methylation frequency (%) and RNAseq coverage (RPGC) in wild-type (WT) and progenies of *epiER*s A5.B1 and A5.C6. Grey box, AT2TE20205 (CACTA1). (**D**) Map of transposon insertion in AT3G11330 and its segregation in *epiER* A5.B1.3 progenies. P1-3, primers used for PCR amplification and sequencing. (**E**) Map of transposon insertion in AT5G10770 and sequence footprint resulting from re-mobilization in *epiER* A5.B1.3 progenies. P4-6, primers used for PCR amplification and sequencing. (**F**) Seed pigmentation defects caused by a sequence insertion in AT5G13930 (*TRANSPARENT TESTA4/CHALCONE SYNTHASE*) resulting from transposon re-mobilization in in *epiER* A5.B1.3 progenies. P7-8, primers used for PCR amplification and sequencing.

We next investigated whether the abnormal distribution of epigenetic marks in *epiER*s could be responsible for the developmental abnormalities observed in these plants. To this end, we performed a RNAseq analysis and found that 1240 and 1128 genes were misregulated in *epiER*s A5.B1 and A5.C6, respectively (Supplementary file 1). A fraction of the upregulated in *epiER*s (483 and 544) were also upregulated in *elf6-C/ref6-5* plants (Figure 5A; Figure 5—figure supplement 1A). Gene ontology analysis revealed that most upregulated genes in the epimutants were involved in biotic stress responses (Figure 5B; Figure 5—figure supplement 1B). When we investigated the chromosomal distribution of these deregulated genes, we found that some were located in constitutive pericentromeric heterochromatin and showed the strongest upregulation effect (Figure 5C). These data suggest that the abnormal distribution of epigenetic marks in *epiERs* results in transcriptional activation of euchromatic and heterochromatic loci. Given that pericentromeric heterochromatin in plants is rich in TEs and is tightly regulated by DNA methylation and other epigenomic modifications (Dubin et al., 2018), we tested whether the epigenomic perturbations found in *epiERs* could result in the activation of transposons. We initially used our transcriptome data to determine the transcriptional state of different TEs in the two *epiER* progenies. We found that both RNA and DNA transposon families were significantly upregulated in *epiERs* (Figure 6A; Figure 6—figure supplement 1). We next determined their copy number in different *epiER* lines (see Materials and methods) to assess whether the transcriptional activation of TEs in these epimutants could result in increased mobility. We found that one heterochromatic transposon, CACTA1 (At2TE20205), and one euchromatic retrotransposon, EVADE (EVD) (At5TE20395), showed a significant increase in copy number in both *epiERs* (Figure 6B). Further analysis revealed that these TEs were depleted in DNA methylation and significantly upregulated (Figure 6C; Figure 6—figure supplement 2). We then determined the precise location of some of the transposons that had newly mobilized in the different epimutants. Most novel insertions accumulated in euchromatin, continued to be active over multiple generations, and sometimes disrupted expression of genes that could be linked to the observed developmental phenotypes (Figure 6D–F and Supplementary file 2). Collectively, our data demonstrate that the developmental abnormalities found in *epiER* lines arise from heritable epigenetic changes and from genetic mutations caused by TE mobilization and reinsertion into the genome.

**Figure 5—figure supplement 1.**
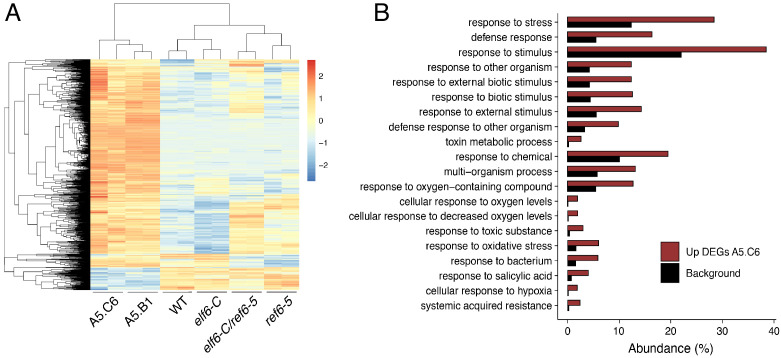
Genes upregulated in *epiERs*. (**A**) Heatmap showing scaled expression levels of Differentially Expressed Genes (DEGs) between wild-type and progeny of epi*ER* A5.C6 in wild-type (WT) *elf6-C*, *ref6-5*, *elf6-C/ref6-5*, and progenies of *epiER*s A5.B1 and A5.C6. (**B**) Gene Ontology analysis showing the functional categories enriched in genes upregulated in *epiER* A5.C6.

**Figure 6—figure supplement 1.**
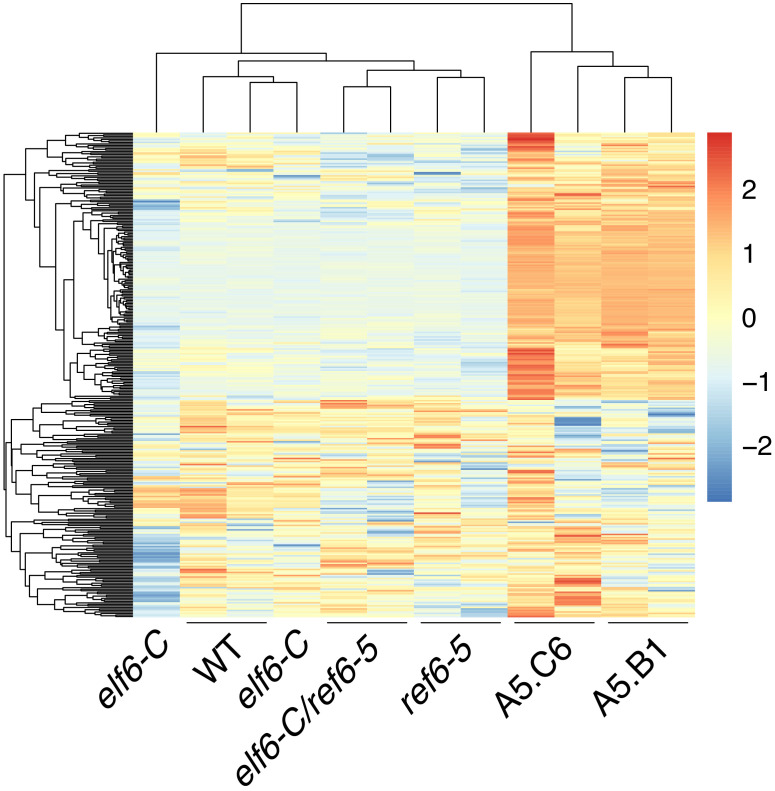
Transcriptional upregulation of transposons in *epiERs*. Heatmap showing scaled logged expression of all the families of transposons or Arabidopsis. Samples represented are wild-type (WT), histone demethylase mutants and *epiERs* A5.B1 and A5.C6.

**Figure 6—figure supplement 2.**
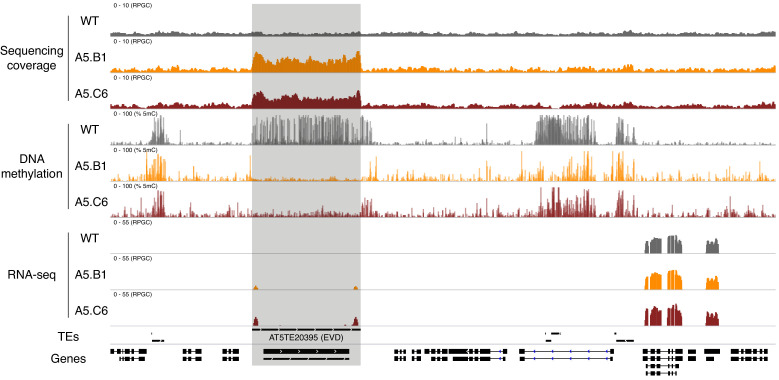
Deregulation of EVD transposon in *epiERs*. Genome browser view showing normalised sequencing coverage (RPGC), DNA methylation rate (%) and RNA-seq (RPGC) in wild-type (WT) and progenies from *epiERs* A5.B1 and A5.C6. Grey box, AT2TE20395 (EVD).

## Discussion

**Figure 7.**
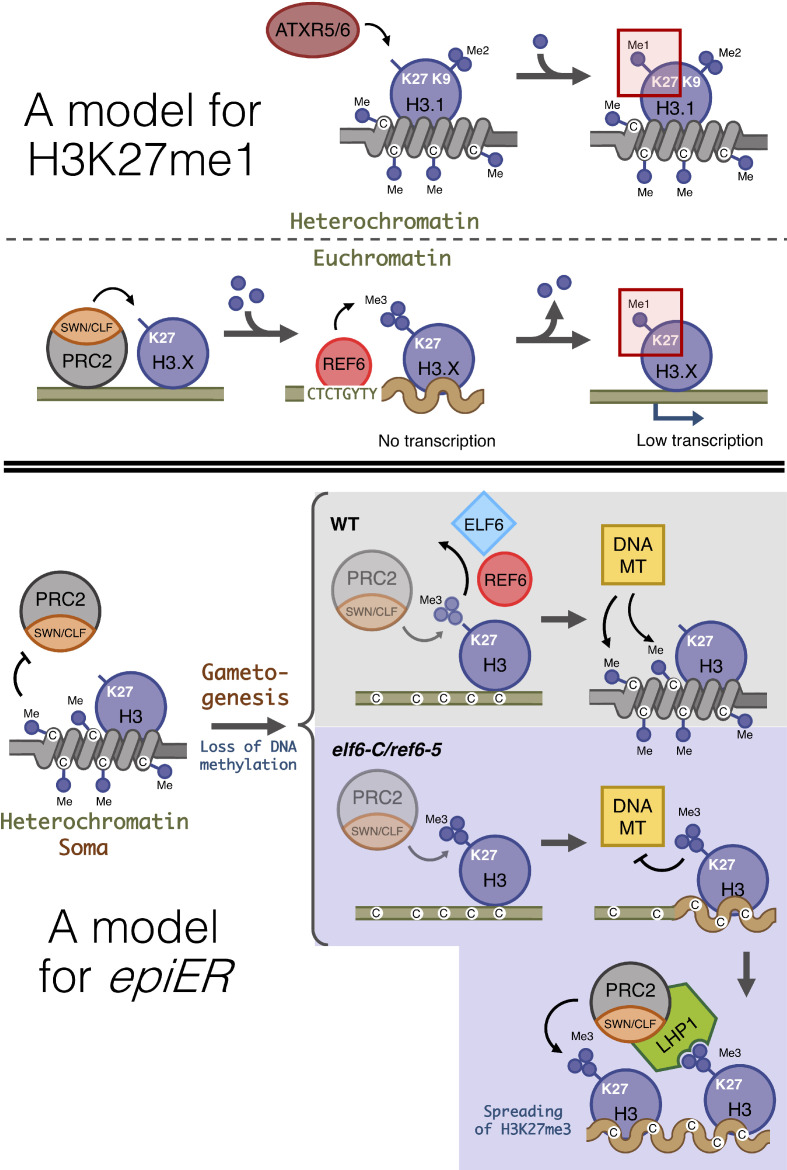
Proposed model for the role of histone demethylases in the accumulation of H3K27me1 and the formation of epimutations arising in ELF6 and REF6 mutants (*epiERs*). (Top panel) Model for the mechanisms implicated in the accumulation of H3K27me1. In pericentromeric heterochromatin of somatic cells, ATRX5/6 deposits in a single-step H3K27me1 on histones containing H3K9me2. In euchromatin of somatic cells, the PRC2 complex deposits H3K27me3, which is converted to H3K27me1 by the catalytic activity of REF6. (Bottom panel) Model for the origin of epiERs. In constitutive heterochromatin of wild type somatic cells, DNA methylation obstructs the PRC2 complex from depositing H3K27me3. In gametes of wild-type plants, reprogramming of DNA methylation facilitates the deposition of H3K27me3 by the PRC2 complex, but these imprints are actively removed by histone demethylases, thus permitting DNA methylases to establish normal levels of methylation. In gametes of elf6-C/ref6-5, the H3K27me3 deposited by the PRC2 complex accumulates during the reprogramming of DNA methylation and interfere with the activity of DNA methyltransferases. The ectopic accumulation of H3K27me3 spreads to flanking genomic regions by recruitment of LHP1 and the PRC2 complex. Dark blue circles, methyl groups. C, Cytosines. H3.X, H3 variant that is not H3.1. Coiled lines represent closed and inactive chromatin. Wavy lines represent Polycomb-repressed chromatin. DNA MT: DNA methyltransferases. Faded shapes represent low amount of enzyme.

In plants, histone modifications deposited by PRC2 play a critical role in growth and development, and in the adaptation of these processes to environmental fluctuations. Previous studies in Arabidopsis have shown that the activity of a distinct group of JmJ-type demethylases shape the genomic distribution of H3K27me3 (Yan et al., 2018). Three of these proteins – JMJ13, ELF6 and REF6 – have been shown to play important roles in development and in the regulation of environmental perception (Noh et al., 2004; Zheng et al., 2019). Our data show that REF6 and ELF6 regulate the removal of H3K27me3 at different genomic loci; while REF6 has a large repertoire of target genes, ELF6 activity is restricted to a small subset of genes, most of which can also be targeted by REF6. Combined with our genetic analysis, these data collectively suggest that despite the structural similarities between these two proteins, they are able to carry out distinct functions in H3K27me3 homeostasis. Our data also support the view that although REF6 restricts the spreading of H3K27me3 to the genomic regions flanking PRC2 targets (Yan et al., 2018), it also has a hitherto unrecognized function in the regulation of H3K27me1 homeostasis in euchromatin. This view is also supported by the overlap between REF6 genomic targets and H3K27me1 accumulation in euchromatic regions in wild-type plants, as well as by the complete loss of H3K27me1 in PRC2 target loci in plants without REF6 activity, and the partial loss of H3K27me1 in plants without PRC2 activity. Therefore, the accumulation of H3K27me1 in Arabidopsis relies both on the activity of ATXR5 and ATXR6 in heterochromatin (Jacob et al., 2009; Jacob et al., 2010) and on the activity of REF6 in transcriptionally active euchromatin (Figure 7). ATXR5 and 6 have been shown to target only newly synthesized H3.1 histone variants, which are deposited during replication and replaced by H3.3 in euchromatin during later stages of the cell cycle (Jacob et al., 2014). In mammals, the histone demethylases UTX and JMJD3, also known as KDM6A and KDM6B, have been shown to catalyze the conversion of H3K27me3 and H3K27me2 into H3K27me1 (De Santa et al., 2007; Lan et al., 2007; Lee et al., 2007; Swigut and Wysocka, 2007). Moreover, defects in PRC2 methyltransferase activity in mammals completely abolishes the accumulation of H3K27me1 in embryonic stem cells (Ferrari et al., 2014; Montgomery et al., 2005), suggesting a conserved PRC2-mediated mechanism for H3K27me1 homeostasis in euchromatin, in both animals and plants. The precise mechanism responsible for the deposition of this chromatin mark in Arabidopsis is currently unknown, but our data support the view that the deposition of H3K27me1 in euchromatin is dependent on the activity of PRC2 and REF6 (Figure 7). In mammals the presence of H3K27me1 in actively transcribed genomic regions has been associated with the promotion of transcription (Ferrari et al., 2014), a fact that may explain why Arabidopsis genes associated with H3K27me1 display moderate levels of expression, whereas the conversion of this mark into H3K27me3 negatively impacts their transcriptional rates.

Plant somatic cells accumulate H3K27me3 primarily at protein-coding genes; however, in reproductive tissues and mutants where DNA methylation is reduced, this mark also accumulates at TEs (Deleris et al., 2012; Weinhofer et al., 2010). Other studies have also reported the accumulation of H3K27me3 at transposon sites in plant species with reduced levels of DNA methylation (Montgomery et al., 2020), as well as in mammal somatic and reproductive tissues which also show reduced levels of DNA methylation (Hanna et al., 2019; Reddington et al., 2014; Saksouk et al., 2014). However, our data do not fully support the idea that the deposition of this chromatin mark acts as a compensatory system to silence hypomethylated TEs (Deleris et al., 2012; Hanna et al., 2019). Instead, our results suggest that the homeostasis and function of H3K27me1 and H3K27me3 in plants is more complex than previously anticipated. The stable inheritance of de novo acquired DNA methylation imprints in plants is well documented. Mutations in the machinery involved in the deposition of DNA methylation, such as the cytosine *DNA METHYLTRANSFERASE 1* (*MET1)* and the chromatin-remodeling *DEFICIENT IN DNA METHYLATION 1* (*DDM1*), frequently induce epimutations caused by DNA hypomethylation (Johannes et al., 2009; Kakutani et al., 1996; Mathieu et al., 2007). These epimutations are maintained during sexual reproduction and remain stable over several generations, even after the function of MET1 or DDM1 is restored. Moreover, natural epimutations created during asexual propagation and associated with DNA hypomethylation involving TEs, can be stable over multiple generations, thus contributing to a variety of novel heritable phenotypes (Ong-Abdullah et al., 2015; Wibowo et al., 2018). Additionally, analysis of the Arabidopsis *met1* mutant has shown that a large number of TEs that lose DNA methylation gain H3K27me3 (Deleris et al., 2012). Similarly, TEs in Arabidopsis gain H3K27me3 in response to *ddm1*-induced loss of DNA methylation (Rougée et al., 2020). Collectively, our data together with these reports suggest that DNA methylation and H3K27me3 act antagonistically to mediate the transcriptional silencing of transposons.

There is accumulating evidence for the active role of histone demethylases in resetting H3K27me3 at specific loci during sexual reproduction (Crevillén et al., 2014; Noh et al., 2004) and for a global depletion of H3K27me3 during spermatogenesis (Borg et al., 2020). While the precise mechanism(s) governing this phenomenon remains poorly understood, our finding that the failure to reset H3K27me3 during sexual reproduction resulted in its trans-generational inheritance in euchromatin, even when functional demethylase activity was restored, might suggest that some of the H3K27me3 imprints that are ectopically deposited in histone demethylase mutants cannot be reset if they are distal to the target sequences recognized by the demethylases. Furthermore, once established, these H3K27me3 imprints could be maintained across generations as epimutations through the recruitment of LHP1-PRC2 complexes (Derkacheva et al., 2013). Our data revealed that the inheritance of H3K27me3 imprints causes defects on the maintenance of DNA methylation at constitutive heterochromatic. However, these defects are not caused by the ectopic accumulation of H3K27me3 at genes implicated in the DNA methylation machinery. The ectopic deposition of H3K27me3 in constitutive heterochromatin may instead be linked to defects in the resetting of DNA methylation thought to take place during gametogenesis (Calarco et al., 2012; Ibarra et al., 2012; Slotkin et al., 2009) and/or during early embryo development (Bouyer et al., 2017), where epigenetic modifications have been shown to be anti-correlative (Mathieu et al., 2005; Roudier et al., 2011). Under this scenario, an active resetting of H3K27me3 in gametes would be critical for the re-establishment of DNA methylation, while the ectopic deposition of H3K27me3 in histone demethylase mutants could antagonize the deposition of DNA methylation at TEs (Figure 7). Mutants defective in histone demethylases could accumulate epigenomic alterations in gametes that could explain the heritable, yet unstable, phenotypes observed in *epiER*s. Similar epimutations and phenotypic variation have been shown to arise from crosses between wild-type plants and mutants defective in the machinery that maintains DNA methylation (Kakutani et al., 1996; Kato et al., 2004; Marí-Ordóñez et al., 2013; Mirouze et al., 2012). As in these studies, we also found that *epiER*s have defects in the silencing of some transposons resulting in an increase in genetic lesions associated with their mobilization. Taken together our data reveal novel, critical roles for histone demethylases in maintaining both genome integrity and transcriptional states during plant development.

## Materials and methods

### Plant material and growth conditions

All plant lines used in this study were derived from *Arabidopsis thaliana,* Col-0 accession. The T-DNA insertion lines *ref6-1* (SALK_001018), *elf6-3* (SALK_074694), *atxr5* (SALK_130607) and *atxr6* (SAIL_240_H01) have been previously described. The *ref6-5* mutant (GABI_705E03) was obtained from the GABI-Kat collection (Kleinboelting et al., 2012) and a genomic deletion line for *elf6-C* was produced using two sgRNAs (Supplementary file 3) and CRISPR/Cas9 (Durr et al., 2018). New mutant alleles were backcrossed twice to wild-type plants and homozygous plants were identified from F_2_ progenies by molecular genotyping (Supplementary file 3). Double mutants between different mutant alleles were produced by manual pollination. The plant materials used for crossing and flowering time measurements were grown in chambers under long day conditions (16 hr light, 8 hr dark) with 120 μ mol m^−2^ s^−1^ light intensity (22°C daytime, 20°C at night). Plants for the screening were grown in a climate-controlled greenhouse under long day conditions (20°C daytime, 20°C at night,16 h light plus 8 hr dark). The seeds were mixed in 0.1% agarose and underwent 2 d cold treatment at 4°C in the dark. After treatment, seeds were directly sown on soil and transferred to growth a chamber or greenhouse.

### Genotyping and phenotyping

Primary transformants were identified using the seed-specific RFP reporter under a Leica MZ-FL III stereomicroscope (Leica Camera AG). Genotyping of CRISPR/Cas9-based mutations and T-DNA insertions were performed using KAPA-Taq (Sigma-Aldrich) following the manufacturer’s instructions. PCR product size was selected using gel electrophoresis and the introduced genetic lesion was determined by sequencing (Figure 1—figure supplementary file 1). The phenotypes of whole plants, leaf number and rosette size were scored at bolting. Silique length measurement were carried out on the 6^th^-15^th^ siliques of main stems, when the last flowers of the inflorescence started producing siliques. The mean value of the 10 siliques represented the silique length of a plant. For embryo analysis, ovules from self-pollinated plants were cleared with a chloral hydrate solution, observed with a light microscope (Zeiss AxioImager A2) and photographed with a digital camera (Zeiss AxioCam HRm).

### ChIP-seq assay

ChIP-seq assays were performed on 14 day-old in vitro shoot seedlings using anti-H3K27me3 (Millipore 07–449) or anti- H3K27me1 (Millipore 07–448), following a procedure modified from Gendrel et al., 2005. Five grams of plantlets were cross-linked in 1% (v/v) formaldehyde at room temperature for 15min. Crosslinking was then quenched with 0.125 M glycine for 5 min. The crosslinked plantlets were ground and nuclei were isolated and lysed in Nuclei Lysis Buffer (1% SDS, 50 mM Tris-HCl pH 8, 10 mM EDTA pH 8). Cross-linked chromatin was sonicated using a water bath Bioruptor UCD-200 (Diagenode, Liège, Belgium) (15 s on/15 s off pulses; 15 times). The complexes were immunoprecipitated with antibodies, overnight at 4°C with gentle shaking, and incubated for 1 hr at 4°C with 40 µL of Protein AG UltraLink Resin (Thermo Scientific). The beads were washed 2 × 5 min in ChIP Wash Buffer 1 (0.1% SDS, 1% Triton X-100, 20 mM Tris-HCl pH 8, 2 mM EDTA pH 8, 150 mM NaCl),2 × 5 min in ChIP Wash Buffer 2 (0.1% SDS, 1% Triton X-100, 20 mM Tris-HCl pH 8, 2 mM EDTA pH 8, 500 mM NaCl), 2 × 5 min in ChIP Wash Buffer 3 (0.25 M LiCl, 1% NP-40, 1% sodium deoxycholate,10 mM Tris-HCl pH 8, 1 mM EDTA pH 8) and twice in TE (10 mM Tris-HCl pH 8, 1 mM EDTA pH 8).ChIPed DNA was eluted by two 15 min incubations at 65°C with 250 μL Elution Buffer (1% SDS, 0.1 M NaHCO_3_). Chromatin was reverse-crosslinked by adding 20 μL of NaCl 5M and incubated over-night at 65°C. Reverse-cross-linked DNA was submitted to RNase and proteinase K digestion, and extracted with phenol-chloroform. DNA was ethanol precipitated in the presence of 20 μg of glycogen and resuspended in 50 μL of nuclease-free water (Ambion) in a DNA low-bind tube. 10 ng of IP or input DNA was used for ChIP-Seq library construction using NEBNext Ultra DNA Library Prep Kit for Illumina (New England Biolabs) according to manufacturer’s recommendations. For all libraries, 12 cycles of PCR were used. The quality of the libraries was assessed with Agilent 2100 Bioanalyzer (Agilent).

### Computational analysis of ChIP-seq

Single-end sequencing of ChIP samples was performed using Illumina NextSeq 500 with a read length of 76 bp. Reads were quality controlled using FASTQC (http://www.bioinformatics.babraham.ac.uk/projects/fastqc/). Trimmomatic was used for quality trimming. Parameters for read quality filtering were set as follows: Minimum length of 36 bp; Mean Phred quality score greater than 30; Leading and trailing bases removal with base quality below 5. The reads were mapped onto the TAIR10 assembly using Bowtie (Langmead, 2010) with mismatch permission of 1 bp. To identify significantly enriched regions, we used MACS2 (Zhang et al., 2008). Parameters for peaks detection were set as follows: Number of duplicate reads at a location:1; mfold of 5:50; q-value cutoff:0.05; extsize 200; broad peak. Visualization and analysis of genome-wide enrichment profiles were done with IGB. Peak annotations such as proximity to genes and overlap on genomic features such as transposons and genes were performed using BEDTOOLS INTERSECT. To identify regions that were differentially enriched in the H3K27me3 or H3K27me1 histone modification between WT and mutants, we used DIFFREPS (Shen et al., 2013) with parameters of pvalue 0,05; z-score cutoff 2; windows 1000 (Supplementary file 4).

### Expression profiling by RNA-seq

Leaf samples were collected from five plants at the 4 week growth stage. Total RNA was extracted using RNeasy Plant Mini Kit (Qiagen) according to manufacturer’s instructions and used to produce libraries using TruSeq RNA library Prep Kit v2 (Illumina). Pooled libraries were sequenced in a NextSeq550 sequencing platform (Illumina). Two biological replicates were generated for each genotype, and at least 20 million reads were produced per replicate.

### Generation of epimutations using histone demethylase mutants

Five independent homozygous *elf6-C*, *ref6-5 elf6-C*/*ref6-5* plants were selected for reciprocal crosses with wild-type plants (Col-0). All the F_1_ progenies were self-pollinated to generate F_2_ seeds that were grown in individual pots. The frequency of developmental phenotypes, not observed in the histone demethylase mutants, was scored 8 week old plants and fertility was determined according to the production of viable seeds. Plants that displayed developmental phenotypes not found in *elf6-C*, *ref6-5 or elf6-C*/*ref6-5* mutants where genotyped by PCR (Supplementary file 3) to determine their zygosity.

### Bisulfite sequencing

Rosette leaves from five 4 week old plants were pooled for each sample. Genomic DNA was extracted with the DNeasy Plant Mini Kit (Qiagen, Germany). DNA libraries were generated using the Illumina TruSeq Nano kit (Illumina, CA, USA). DNA was sheared to 350 bp. The bisulfite treatment step using the Epitect Plus DNA Bisulfite Conversion Kit (Qiagen, Germany) was inserted after the adaptor ligation; incubation in the thermal cycler was repeated once before clean-up. After clean-up of the bisulfite conversion reaction, library enrichment was done using Kapa Hifi Uracil+ DNA polymerase (Kapa Biosystems, USA). Libraries were sequenced with 2 × 150 bp paired-end reads on an HiSeq 4000 (Illumina), with conventional gDNA libraries in control lanes for base calling calibration. Sixteen to 24 libraries with different indexing adapters were pooled in each lane.

### Computational analysis of paired end BS-seq

Paired-end quality was assessed using FASTQC (Andrews et al., 2010). Trimmomatic (Bolger et al., 2014) was used for quality trimming. Parameters for read quality filtering were set as follows: Minimum length of 40 bp; sliding window trimming of 4 bp with required Phred quality score of 20. Trimmed reads were mapped to the *Arabidopsis thaliana* TAIR10 genome assembly using bwa-meth (Pedersen et al., 2014) with default parameters. Mapped reads were deduplicated using picardtools (Picard toolkit, 2019), and numbers of methylated/unmethylated reads per position were retrieved using MehtylExtract (Oliver et al., 2014).

### Pericentromeric heterochromatic regions

﻿Heterochromatin regions were defined as in Qiu et al., 2019 (Chr1:12,500,000–17,050,000, Chr2:2,300,000–6,300,000, Chr3: 12,800,000–14,800,000, Chr4: 1,620,000–2,280,000; 2,780,000–5,804,000, Chr5: 10,680,000–14,000,000).

### Gene expression and ontology analysis

We used agriGO v2.0 (Tian et al., 2017) to classify significantly enriched Gene Ontology (GO) terms associated with differential expression.

### Immunostaining of chromatin

Leaf protoplasts were isolated from 14 day old seedlings and fixed. After rehydration in PBS, slides were blocked in 2% BSA in PBS (30 min, 37°C) and incubated overnight at 4°C in 1% BSA in PBS containing antibodies (Upstate Biotechnology) specific to lysine-27-monomethylated H3 (1:100 dilution), and lysine-27-trimethylated H3 (1:100 dilution). Detection was carried out with an FITC-coupled antibody to rabbit IgG (Molecular Probes; 1:100 dilution, 37°C, 40 min) in 0.5% BSA in PBS. DNA was counterstained with 4,6 diamidino-2-phenylindole (DAPI) in Vectashield (Vector Laboratories).

### Data visualisation

﻿For visualising BS-seq, RNA-seq and ChIP-seq genomic data we used Integratice Genomic Viewer (IGV) (Thorvaldsdóttir et al., 2013), And R version 3.5.1 (www.r-project.org) with packages ggplot2 (Wickham, 2016), eulerr (Larsson, 2019), pheatmap (Kolde, 2015) and EnrichedHeatmap (Gu et al., 2018).

### Prediction of new TE insertion sites and molecular validation

We analysed Bisulfite-seq data using Bismark (Krueger and Andrews, 2011) using the following parameters:–bowtie2 –ambiguous –unmapped –R 10 –score_min L,0,–0.6 -N 1. Identification of new TE insertion sites was performed using epiTEome (Daron and Slotkin, 2017). For the validation of new transposon insertions, we designed primers outside of predicted TE insertion site and inside the transposon based on physical reads identified by epiTEome. We used KAPA Taq Polymerase and PCR conditions of 95°C for 5 min, followed by 30–35 cycles of 95°C for 30 s, 58°C for 15 s, and 72°C for 2 min. The list of primers employed for this analysis are listed (Supplementary file 3).

### Major datasets

The following dataset was generated: ‘Arabidopsis H3K27 demethylases contribute to genomic integrity’. Dataset URL https://www.ebi.ac.uk/ena/browser/view/PRJEB36508.

The following previously published datasets were used: DDM1 and RdDM are the major regulators of transposon DNA methylation in Arabidopsis’. Dataset URL https://www.ncbi.nlm.nih.gov/geo/query/acc.cgi?acc=GSE41302.

## Data Availability

Sequence data (BS-seq, RNA-seq and ChiP-seq) that support the findings of this study have been deposited at the European Nucleotide Archive (ENA) under the accession code PRJEB36508. The following dataset was generated: SanchezJA2020Arabidopsis H3K27 demethylases contribute to genomic integrityEuropean Nucleotide ArchivePRJEB36508 The following previously published datasets were used: QiuQ2019DNA methylation prevents REF6 binding in ArabidopsisNCBI Gene Expression OmnibusGSE111830 ZemachA2013DDM1 and RdDM are the major regulators of transposon DNA methylation in ArabidopsisNCBI Gene Expression OmnibusGSE41302

## Additional files

Supplementary file 1. Misregulated genes in *epiERs*.

Supplementary file 2. New TE insertion sites predicted by epiTEome in *epiERs*.

Supplementary file 3. Oligonucleotides used to genotype genetic lesions in mutants and *epiERs*.

Supplementary file 4. ChIP peaks and associated genes.

Transparent reporting form
