# Supplementary material for: A new role for histone demethylases in the maintenance of plant genome integrity
Source: eLife. 2020 Oct 27;9:e58533. doi: 10.7554/eLife.58533 (PMC7671693; doi:10.7554/eLife.58533)
Supplement: Peer review history

# Decision letter

**DOI:** 10.7554/eLife.58533.sa1

**Affiliations:** Seoul National UniversityRepublic of Korea

In the interests of transparency, eLife publishes the most substantive revision requests and the accompanying author responses.

**Acceptance summary:**

This study illustrates a novel role of H3K27me2/3 demethylases in homeostasis of H3K27me1 level in plants. It is also noteworthy that resetting the chromatin marks is particularly important for maintaining genetic and epigenetic stability in the next generation.

**Decision letter after peer review:**

Thank you for submitting your article "A new role for histone demethylases in the maintenance of plant genome integrity" for consideration by *eLife*. Your article has been reviewed by three peer reviewers, one of whom is a member of our Board of Reviewing Editors, and the evaluation has been overseen by Christian Hardtke as the Senior Editor. The following individual involved in review of your submission has agreed to reveal their identity: Yannick Jacob (Reviewer #2).

The reviewers have discussed the reviews with one another and the Reviewing Editor has drafted this decision to help you prepare a revised submission.

We would like to draw your attention to changes in our revision policy that we have made in response to COVID-19 (https://elifesciences.org/articles/57162). Specifically, when editors judge that a submitted work as a whole belongs in *eLife* but that some conclusions require a modest amount of additional new data, as they do with your paper, we are asking that the manuscript be revised to either limit claims to those supported by data in hand, or to explicitly state that the relevant conclusions require additional supporting data.

Our expectation is that the authors will eventually carry out the additional experiments and report on how they affect the relevant conclusions either in a preprint on bioRxiv or medRxiv, or if appropriate, as a Research Advance in *eLife*, either of which would be linked to the original paper.

All reviewers agree that this work looks interesting, but all three reviewers also raised major issues about biological relevance of H3K27me1 in plants and mechanisms behind epimutation and linkages of DNA methylation and H3K27me3. Reviewer 1 and 3 also critisized that paper has two main findings, which are not cohesive. Essential revisions:

1) The most important issue is how ectopic accumulation of H3K27me3 leads to impaired DNA methylation. The mechanistic linkage is poor in the current manuscript, and this should be substantialized in the revision.

2).Please address the biological relevance of H3K27me1, which is not clear. It could be indirectly linked to gene expression control and genome stability.

3) The paper could be separated. The first part covers a new biochemical function of REF6 and ELF6, but the second part mainly deals with epimutation in progenies. It is not clear that changes in H3K27me1/H3K27me3 are relevant for the phenotypic and epigenetic alterations in next generations. The manuscript should be more connected.

However, please thoroughly check the reviewer comments (you find the individual reviews below) and address them to the best of your capacity. All comments are critical for publication in *eLife*.

Reviewer #1:

The contribution by Antunez-Sanchez et al. demonstrates that histone demethylases REF6 and ELF6 have distinct roles in the histone demethylation in Arabidopsis. REF6 has a broader impact in reducing H3K27me3 and is also a major player in the deposition of H3K27me1 in active euchromatin regions. The maintenance of H3K27me1 in active chromatin tends to allow a low-level basal expression. In addition, this study also showed that the failure to reset H3K27me marks during sexual reproduction results in the inheritance of H3K27me3 imprints, which is associated with the loss of DNA methylation at heterochromatic loci, leading to TE activations. Overall, the manuscript covers new roles of REF6 and ELF6 and biological relevance of H3K27me1/me3 homeostasis in genome integrity and transcription activity. However, several flaws should be addressed to show the importance of this study.

1) What tissues were used for each analysis? Genetic interactions between ELF6 and REF6 vary as shown in phenotypic analysis. Therefore, plant tissues and age should be described in figure legends.

2) They suggested the importance of H3K27me1 homeostasis in gene expression. I am wondering if the H3K27me1 imprints can be transmitted to offspring in wild type. Otherwise, how the histone mark could be reset. Is there another responsible histone demethylase that removes H3K27me1? In addition, the relevance of H3K27me1 in genome stability is limited, and interpretation about epi-mutants relies mainly on ectopic accumulation of H3K27me3. Although they emphasize their finding about REF6-catalyzed H3K27me1 as shown in Figure 7, the analysis of H3K27me1 was not extensive.

3) Do REF6 and ELF6 have critical roles in silencing pericentromoric regions? How about H3K27me3 and H3K27me1 levels in pericentromeric regions in ref6 and elf6 mutants?

4) Ectopic accumulation of H3K27me3 leads to global reductions in DNA methylation at pericentromeric regions. To support the idea that hypomethylation caused by ectopic accumulation of H3K27me3 depends on RdDM pathways, they should use mutants of RdDM pathways. In addition, if REF6 and ELF6 are particularly relevant in histone modification at active chromatin, what mechanisms could be involved in changes in DNA methylation at pericentromeric regions? I think that DNA methylation could be indirect and stochastic results.

5) Reduction of H3K27me1 could be linked to global reductions in DNA methylation? There is no experiment to rule out the possibility.

6) Individual epi-mutants have variable phenotypes, and genetic backgrounds are not likely uniform. I am wondering if GO analysis is meaningful in this situation. It could be very different depending on individuals.

7) Figure 4E: Please quantify the results, rather than showing representative images.

8) Figure 7: The summary figure should show main finding of this paper. I think only the fraction of this study is shown, and this makes reader confusing. The authors should rearrange the manuscript and figures and give a more focused view of the conclusion.

Reviewer #2:

General Assessment

The manuscript by Antunez and colleagues provide important biological insights into the role of DNA demethylase in maintaining genetic and epigenetic stability between different generations. In addition, this study also shed light on the contribution of H3K27me2/3 demethylases toward in vivo levels of H3K27me1 in plants. I think this is important work that should be of general interest to the plant epigenetic community. The manuscript is well-written and the conclusions are substantiated.

Substantive concern:

1) One concern with the manuscript is the mechanism leading to epimutations that is proposed by the authors, which is that ectopic inheritance of H3K27me3 in gametes leads to the different phenotypes observed. The work of Olivier Mathieu and his colleagues in 2005 has shown that loss of DNA methylation in ddm1 mutants leads to ectopic gains of H3K27me3 in heterochromatin. Transcriptional reactivation of transposons and transposition (e.g. Tsukahara et al., Nature 2009) is also observed in ddm1 mutants like in the EpiER lines. Therefore, it would be useful for the authors to investigate if DNA methylation is impaired because of genome-wide gains in H3K27me3 in gametes, or whether one or a few affected loci are responsible for the loss of DNA methylation. The authors try to address this point (e.g. subsection “Accumulation of ectopic H3K27me3 at centromeric heterochromatin is linked to DNA hypomethylation”), however, more information should be provided. They should indicate which genes in the DNA methylation pathways have been assessed for H3K27me3 levels. In addition, the transcriptional status of these genes should be assessed (the data is already available from this study) to determine if loss of DNA methylation is due to down-regulation of DNA methylation genes. Transcriptional silencing of these genes could be achieved indirectly, if no gains in H3K27me3 are observed at the genes.

2) Results: It's not clear to me looking at Figure 2E and Figure 2—figure supplement 5 that REF6 is "…required for low-level expression genes." I think those figures indicate that these genes (3rd and 5th quantiles) gains H3K27me3, but the transcriptional impact of the increase of H3K27me3 at these genes is not provided. Since they have transcriptional data for ref6 mutants, the authors could look if gains in H3K27me3 at these genes decrease their expression.

Reviewer #3:

The paper looks at the effects of the Arabidopsis H3K27me3 histone demethylases (HDM) REF6 and ELF6 on the plant epigenome and on the generation of novel epialleles. There are two fairly distinct parts to the paper. In the first part, they generate elf6 ref6 double mutants using likely null alleles, one generated by genome editing. They use genomic approaches to show that many target genes gain H3K27me3 methylation and have decreased expression in the mutants. This part is consistent with what has been shown by several other studies, notably the Kaufman group ( Nature Plants, 4:681). Of greater novelty, they then show that REF6/ELF6 targets are enriched for H3K27me1 in the wild type background, but lose this mark in ref6 elf6 double mutants, consistent with a role for REF6 and ELF6 in generating this mark by demethylating H3K27me3 to H3K27me1 but being unable to demethylate H3K27me1 substrates. Using ChIP seq they show that ATXR5 and ATXR6 (H3K27me1 monomethylases required for heterochromatic H3K27me1) are not required for the H3K27me1 that they find at euchromatic locations. They correlate H3K27me1 in euchromatin with intermediate level gene expression, although whether this is an indirect effect of a lack of H3K27me3 is hard to disentangle. In the second part they describe the appearance of novel phenotypes at low frequency in F2 progeny of WT X elf6 ref6. They identify REF6+ ELF6+ homozygous progeny in F2 and show that these can transmit novel phenotypes from F2 to F5 generations. Profiling of selected F5 individuals with novel phenotypes reveal various changes, including H3K27me3 hyper and hypomethylation, loss of DNA methylation, and mobilisation of transposable elements (TE). The suggestion is that the novel phenotypes are caused by epialleles, i.e. heritable alterations in histone or DNA methylation, although DNA sequence based changes due to TE insertion or imprecise excision are also possible causes.

Overall I found this a very interesting paper. The role of HDM in euchromatic H3K27me1 is novel and intriguing, perhaps difficult to assess the significance as the mechanism of H3K27me1 action is not known in plants, i.e. readers etc for this mark have not been identified. The observation that ref elf mutants may give rise to epialleles that persist even when REF6 ELF6 activity is restored is of broad interest, and builds on studies such as Crevillen et al. (Nature 515: 587) which showed that elf6 impairment can give rise to heritable epigenetic changes (but in this case only in the elf6 mutant background). On the negative side, the two parts of the paper are not very strongly connected, for example it is not very clear if changes in H3K27me1 described in part one are relevant for the phenotypic and epigenetic alterations described in part two. Also, there is very limited description of the phenotypes found and the causes are not very clear i.e. alterations in which genes are causal and what are the genetic or epigenetic changes involved? It is not very clear why ref6/elf6 depletion gives rise to the effects observed, for example one of the major changes in hyper H3K27me3 methylation and loss of DNA methylation at pericentromeric heterochromatin, yet ref6 elf6 mutants do not seem to show this change. Overall, the observations are interesting, they have done a lot of analysis and it is unreasonable to expect a complete explanation but the overall picture remains confusing. As such I think the paper is borderline for *eLife*, and would at least require some revision along the lines below.

1) Figure 3A shows plants with aberrant phenotypes and their transmission, however there is no description of the phenotypes, or of their inheritance i.e. what kind of segregation ratios are observed in the different generations. For example, A5 looks to be broad leaved but gives rise to plants with narrow curled leaves in F3 generation, it is not clear if these are transmitted into F4 generation or at what frequency, or if traits are dominant or recessive.

2) It's not very clear which generation is used for the various profiles of A5 progeny, possibly F4 or F5 plants?

3) Much of the data is not accessible or presented. For example, no lists of the peaks found for the different marks profiled are provided, nor are there lists of differential peaks found, or how these related to genes showing altered expression.

4) In Figure 3D the wild type trace is invisible, either omitted or masked by another trace?

5) The recent paper from Berger group on reprogramming in sperm (Borg et al., 2020) could be referenced and discussed. This study would suggest that H3K27me3 hypermethylation should not be transmitted paternally once REF6/ELF6 activity is restored. It would be interesting to know if the phenotypes and or epigenetic changes seen in A5 can be transmitted maternally, paternally or both once RER6/ELF6 activity is restored. Given that some or all of the effects of REF6/ELF6 depletion may be indirect effects of H3K27me3 changes, for example TE mobilisation or DNA hypomethylation, it is possible that the phenotypes would be transmitted but this might give some indication of whether H3K27me3 changes are causal for the phenotypes or not, or indirect. If the phenotypes are recessive, then this would require both paternal and maternal transmission in self progeny.

6) Subsection “REF6 controls H3K27me1 homeostasis in chromatin” states that H3K27me1 is not affected at euchromatic gene in atxr5 atxr6 mutants, however in Figure 2—figure supplement 2 it looks as if levels are actually increased in the mutant. This should be commented on and discussed. It should also be mentioned that the particular atxr5 atxr6 double mutant combination is hypomorphic and not null.

7) Figure 6F shows that line A5.B1.3 which has a seed pigmentation phenotype has a 5 bp insertion in the tt4 gene. It is stated that this is due to transposon remobilisation but it is not clear if this is inferred as a likely imprecise excision, or whether they are able to show that a TE is present in this location in a progenitor plant?

8) Given that a lot of sequence data is available for some of the lines, I wondered if authors should check that there are no mutations present in genes associated with RdRM etc? That is not intended as a slight, but since changes in DNA methylation are observed that don't readily correlate with what is seen in the ref6 elf6 parental lines it could presumably be easily checked and eliminated as a potential cause.

9) The correlation between pericentromeric H3K27me3 increase and DNA methylation decrease is an interesting observation, but they provide no mechanism why this should arise in elf6 ref6 backgrounds, which they show seem normal for pericentromeric DNA methylation and H3K27me3. The Qiu et al. (2019) Nat Comm paper, which the authors here mention in the Introduction, reported that REF6 cannot bind efficiently on methylated DNA, so it is puzzling.

10) Lines were picked based on aberrant phenotypes, propagated, and then analysed and found to contain various epigenetic and genetic changes. It is difficult to know whether the changes cause the phenotypes seen, and if so which particular changes. This made me wonder to what extent ELF6 REF6 F2 progeny with normal phenotypes would also show epigenomic changes, or whether this is specific to the plants with phenotypic abnormalities?

11) The fact that ref6 and elf6 have opposite effects on flowering time is curious. The elf6c mutant is early flowering, consistent with a role for ELF6 in binding the floral repressor FLC and promoting its expression by H3K27me3 demethylation (Mol Plant 11:1135). The ref6 mutant is late flowering, and has been correlated with increased FLC expression (Plant Cell 16: 2601), which seems at odds with the hypermethylation for H3K27me3 and decreased expression more typical of these mutants. This might be commented on, and whether the various profiling experiments offer any explanation.

[Editors' note: further revisions were suggested prior to acceptance, as described below.]

Thank you for submitting your article "A new role for histone demethylases in the maintenance of plant genome integrity" for consideration by *eLife*. Your article has been reviewed by three peer reviewers, and the evaluation has been overseen by Christian Hardtke as the Senior Editor. The following individual involved in review of your submission has agreed to reveal their identity: Yannick Jacob (Reviewer #2).

The reviewers have discussed the reviews with one another and the Reviewing Editor has drafted this decision to help you prepare a revised submission.

We would like to draw your attention to changes in our revision policy that we have made in response to COVID-19 (https://elifesciences.org/articles/57162). Specifically, we are asking editors to accept without delay manuscripts, like yours, that they judge can stand as *eLife* papers without additional data, even if they feel that they would make the manuscript stronger. Thus the revisions requested below only address clarity and presentation.

Reviewer #3:

The authors have provided some new data, ChIP seq of H3K27me1 in clf swn mutants, which provides some support for their model that euchromatic H3K27me1 derives from demethylation by REF6 of H3K27me3 deposited by PRC2 and is a good addition. Overall I find it an interesting paper of broad general interest but don't feel that the revision greatly addresses the comments from before as to mechanism and significance of H3K27me1 being unclear. That said, there is a lot of data in the paper and it is an interesting story that is perhaps complementary to the Borg et al., 2020 paper.

I have issues with a couple of the points made by authors.

3) Much of the data is not accessible or presented. For example, no lists of the peaks found for the different marks profiled are provided, nor are there lists of differential peaks found, or how these related to genes showing altered expression. All the raw data has been uploaded in a public repository. We don't think it is necessary to upload all of the peak data as supplemental information.

It is a given that the raw data will be provided. Since there is a lot of discussion of the ChIP seq data and selected peaks etc are shown I think it reasonable that the authors provide lists of enriched genes for the various ChIP seq experiments, as is usually provided in other papers.

8) Given that a lot of sequence data is available for some of the lines, I wondered if authors should check that there are no mutations present in genes associated with RdRM etc? That is not intended as a slight, but since changes in DNA methylation are observed that don't readily correlate with what is seen in the ref6 elf6 parental lines it could presumably be easily checked and eliminated as a potential cause.

We have investigated the chromatin (H3K27me3 and H3K27me1) and expression profile of gene implicated in DNA methylation genes, both in our histone demethylase mutants and also in epiERs (see heatmap in response to reviewer 2). We only found one differentially expressed gene (FDM2) that acts redundantly with five other genes.

This does not answer the question. A mutation in one of the RdRM genes will not necessarily alter expression level (nonsense mediated decay could occur, but not necessarily) or chromatin.

# Reply: Author response

**DOI:** 10.7554/eLife.58533.sa2

Reviewer #1:

The contribution by Antunez-Sanchez et al. demonstrates that histone demethylases REF6 and ELF6 have distinct roles in the histone demethylation in Arabidopsis. REF6 has a broader impact in reducing H3K27me3 and is also a major player in the deposition of H3K27me1 in active euchromatin regions. The maintenance of H3K27me1 in active chromatin tends to allow a low-level basal expression. In addition, this study also showed that the failure to reset H3K27me marks during sexual reproduction results in the inheritance of H3K27me3 imprints, which is associated with the loss of DNA methylation at heterochromatic loci, leading to TE activations. Overall, the manuscript covers new roles of REF6 and ELF6 and biological relevance of H3K27me1/me3 homeostasis in genome integrity and transcription activity. However, several flaws should be addressed to show the importance of this study.

1) What tissues were used for each analysis? Genetic interactions between ELF6 and REF6 vary as shown in phenotypic analysis. Therefore, plant tissues and age should be described in figure legends.

In the revised Materials and methods, we have included information about the age and type of plant tissues used.

2) They suggested the importance of H3K27me1 homeostasis in gene expression. I am wondering if the H3K27me1 imprints can be transmitted to offspring in wild type. Otherwise, how the histone mark could be reset. Is there another responsible histone demethylase that removes H3K27me1? In addition, the relevance of H3K27me1 in genome stability is limited, and interpretation about epi-mutants relies mainly on ectopic accumulation of H3K27me3. Although they emphasize their finding about REF6-catalyzed H3K27me1 as shown in Figure 7, the analysis of H3K27me1 was not extensive.

The reviewer makes an interesting point. Although it is not yet known how removal of H3K27me1 occurs and which proteins are involved in this process, it has been recently proposed that this might occur during replacement by the histone H3.10 variant (Borg et al., 2020).

We have investigated the relationship between H3K27me3, H3K27me1 and DNA methylation in epiER plants and found a negative correlation between H3K27me3 and the other two epigenetic marks (see heatmap analysis in Author response image 1). We do not think these results affect our current hypothesis but are happy to include these data if deemed necessary.

**Author response image 1.**

3) Do REF6 and ELF6 have critical roles in silencing pericentromoric regions? How about H3K27me3 and H3K27me1 levels in pericentromeric regions in ref6 and elf6 mutants?

Analysis of H3K27me3 and H3K27me1 in ref6-5, elf-C and elf6-C/*ref6-5* did not reveal significant changes in pericentromeric regions compared to WT, thus indicating that these histone demethylases do not play a major role in the regulation of chromatin at these genomic regions. Moreover, the binding of these histone demethylases to chromatin is thought to be prevented by DNA methylation (Qui et al., 2019), therefore their role in silencing pericentromeric regions in somatic tissues appears to be limited.

4) Ectopic accumulation of H3K27me3 leads to global reductions in DNA methylation at pericentromeric regions. To support the idea that hypomethylation caused by ectopic accumulation of H3K27me3 depends on RdDM pathways, they should use mutants of RdDM pathways. In addition, if REF6 and ELF6 are particularly relevant in histone modification at active chromatin, what mechanisms could be involved in changes in DNA methylation at pericentromeric regions? I think that DNA methylation could be indirect and stochastic results.

We thank the reviewer for this comment. We clarified these points in the revised Discussion and explained briefly below:

Deleris et al., 2012 have shown that in the Arabidopsis *met1* mutant impaired for CG methylation, hundreds of TEs that lost DNA methylation also gained H3K27me3. Recently, Rougée et al. (2020) have also shown that numerous TEs gain H3K27me3 in response to *ddm1*-induced loss of DNA methylation. Collectively, these studies suggest that DNA methylation can antagonize H3K27me3 deposition at TEs and that transposon sequences can be marked by H3K27me3 through the activity of the PRC2 complex. In addition, our data show that a gain on H3K27me3 can induce a loss of DNA methylation at TEs. Collectively, our data combined with these reports suggest that these two epigenetic pathways act antagonistically at TE sites. The precise mechanism(s) implicated in changes in DNA methylation at pericentromeric regions are not yet fully understood but we hypothesize that these histone demethylases may be required to protect DNA demethylated regions from the activity of the PRC2 complexes during the reprogramming of heterochromatic marks thought to take place during plant gametogenesis.

5) Reduction of H3K27me1 could be linked to global reductions in DNA methylation? There is no experiment to rule out the possibility.

H3K27me1 is linked to DNA methylation primarily in centromeric and pericentromeric transposon-related sequences but not on euchromatin. Our data shows that the accumulation of H3K27me3 and reduction of H3K27me1 in *ref6-5* mutant is primarily on genic regions (REF6 targets) usually lacking DNA methylation. For these reasons, we did not consider it necessary to perform the experiment suggested by the reviewer.

6) Individual epi-mutants have variable phenotypes, and genetic backgrounds are not likely uniform. I am wondering if GO analysis is meaningful in this situation. It could be very different depending on individuals.

We believe the GO analysis is informative because if specific gene networks are affected in epimutants, which differ in their epigenetic background, it suggests that the genomic regions affected are non-random.

7) Figure 4E: Please quantify the results, rather than showing representative images.

Quantification of nuclear chromatin condensations was provided in Figure 4—figure supplement 3.

8) Figure 7: The summary figure should show main finding of this paper. I think only the fraction of this study is shown, and this makes reader confusing. The authors should rearrange the manuscript and figures and give a more focused view of the conclusion.

We have revised the Discussion and summary figure (Figure 7) to highlight the two main findings of our study.

Reviewer #2:

General Assessment

The manuscript by Antunez and colleagues provide important biological insights into the role of DNA demethylase in maintaining genetic and epigenetic stability between different generations. In addition, this study also shed light on the contribution of H3K27me2/3 demethylases toward in vivo levels of H3K27me1 in plants. I think this is important work that should be of general interest to the plant epigenetic community. The manuscript is well-written and the conclusions are substantiated.

Substantive concern:

1) One concern with the manuscript is the mechanism leading to epimutations that is proposed by the authors, which is that ectopic inheritance of H3K27me3 in gametes leads to the different phenotypes observed. The work of Olivier Mathieu and his colleagues in 2005 has shown that loss of DNA methylation in ddm1 mutants leads to ectopic gains of H3K27me3 in heterochromatin. Transcriptional reactivation of transposons and transposition (e.g. Tsukahara et al., Nature 2009) is also observed in ddm1 mutants like in the EpiER lines. Therefore, it would be useful for the authors to investigate if DNA methylation is impaired because of genome-wide gains in H3K27me3 in gametes, or whether one or a few affected loci are responsible for the loss of DNA methylation. The authors try to address this point (e.g. subsection “Accumulation of ectopic H3K27me3 at centromeric heterochromatin is linked to DNA hypomethylation”), however, more information should be provided.

Our data support the view that DNA methylation is impaired because gametes gain H3K27me3. This hypothesis is supported by a recent report that has shown that histone demethylase mutants cause a significant increase in H3K27me3 in male gametes (Borg et al., 2020).

They should indicate which genes in the DNA methylation pathways have been assessed for H3K27me3 levels. In addition, the transcriptional status of these genes should be assessed (the data is already available from this study) to determine if loss of DNA methylation is due to down-regulation of DNA methylation genes. Transcriptional silencing of these genes could be achieved indirectly, if no gains in H3K27me3 are observed at the genes.

We have checked the chromatin (H3K27me3 and H3K27me1) and expression profiles of genes implicated in DNA methylation in our histone demethylase mutants and in two epiERs. We only found one common differentially expressed gene (FDM2), which is thought to act redundantly with five other genes (FDM1, FDM3, FDM4 and FDM5) in RNAdirected DNA methylation (Xie et al., 2012). Moreover, although this gene is downregulated in *ref6-5* and elf6-C/ref6-5, it does not cause significant changes in DNA methylation.

**Author response image 2.**

2) Results: It's not clear to me looking at Figure 2E and Figure 2—figure supplement 5 that REF6 is "…required for low-level expression genes." I think those figures indicate that these genes (3rd and 5th quantiles) gains H3K27me3, but the transcriptional impact of the increase of H3K27me3 at these genes is not provided. Since they have transcriptional data for ref6 mutants, the authors could look if gains in H3K27me3 at these genes decrease their expression.

When we select the genes hypermethylated at H3K27me3 in *ref6-5* mutants and split them by decile of expression in WT, we observed a significant decrease (Bonferroni adjusted p-value < 0.0001) in the expression between WT plants and *ref6-5* mutants for all deciles; deciles 3 to 5 included.

**Author response image 3.**

Reviewer #3:

[…]

Overall I found this a very interesting paper. The role of HDM in euchromatic H3K27me1 is novel and intriguing, perhaps difficult to assess the significance as the mechanism of H3K27me1 action is not known in plants, i.e. readers etc for this mark have not been identified. The observation that ref elf mutants may give rise to epialleles that persist even when REF6 ELF6 activity is restored is of broad interest, and builds on studies such as Crevillen et al. (Nature 515: 587) which showed that elf6 impairment can give rise to heritable epigenetic changes (but in this case only in the elf6 mutant background). On the negative side, the two parts of the paper are not very strongly connected, for example it is not very clear if changes in H3K27me1 described in part one are relevant for the phenotypic and epigenetic alterations described in part two. Also, there is very limited description of the phenotypes found and the causes are not very clear i.e. alterations in which genes are causal and what are the genetic or epigenetic changes involved? It is not very clear why ref6/elf6 depletion gives rise to the effects observed, for example one of the major changes in hyper H3K27me3 methylation and loss of DNA methylation at pericentromeric heterochromatin, yet ref6 elf6 mutants do not seem to show this change. Overall, the observations are interesting, they have done a lot of analysis and it is unreasonable to expect a complete explanation but the overall picture remains confusing. As such I think the paper is borderline for eLife, and would at least require some revision along the lines below.

1) Figure 3A shows plants with aberrant phenotypes and their transmission, however there is no description of the phenotypes, or of their inheritance i.e. what kind of segregation ratios are observed in the different generations. For example, A5 looks to be broad leaved but gives rise to plants with narrow curled leaves in F3 generation, it is not clear if these are transmitted into F4 generation or at what frequency, or if traits are dominant or recessive.

We have added a description of the phenotypes observed and the ratios at which they appear. We have also added a figure (Figure 3—figure supplement 1 phenotypes of segregating plants).

2) It's not very clear which generation is used for the various profiles of A5 progeny, possibly F4 or F5 plants?

We have clarified in the text that F5 plants were used for the ChIP-seq, RNA-seq and bulk BS-seq, and that F4 plants were used for the BS-seq of individual plants.

3) Much of the data is not accessible or presented. For example, no lists of the peaks found for the different marks profiled are provided, nor are there lists of differential peaks found, or how these related to genes showing altered expression.

All the raw data has been uploaded in a public repository. We don’t think it is necessary to upload all of the peak data as supplemental information.

4) In Figure 3D the wild type trace is invisible, either omitted or masked by another trace?

Figure 3D represents the difference in H3K27me3 levels between the epiER lines and WT plants, 0 in the y axis represents no difference to WT. We have changed the y axis label to clarify this.

5) The recent paper from Berger group on reprogramming in sperm (Borg et al., 2020) could be referenced and discussed. This study would suggest that H3K27me3 hypermethylation should not be transmitted paternally once REF6/ELF6 activity is restored. It would be interesting to know if the phenotypes and or epigenetic changes seen in A5 can be transmitted maternally, paternally or both once RER6/ELF6 activity is restored. Given that some or all of the effects of REF6/ELF6 depletion may be indirect effects of H3K27me3 changes, for example TE mobilisation or DNA hypomethylation, it is possible that the phenotypes would be transmitted but this might give some indication of whether H3K27me3 changes are causal for the phenotypes or not, or indirect. If the phenotypes are recessive, then this would require both paternal and maternal transmission in self progeny.

We have discussed the data that was published by Borg et al., 2020 while our manuscript was under review. Their data support our view that the accumulation of H3K27me3 could have profound consequences in the reprogramming of other epigenetic marks. Our genetic data suggest that histone methylases are required in the male and female germline to prevent the accumulation of epigenetic defects in the offspring.

6) Subsection “REF6 controls H3K27me1 homeostasis in chromatin” states that H3K27me1 is not affected at euchromatic gene in atxr5 atxr6 mutants, however in Figure 2—figure supplement 2 it looks as if levels are actually increased in the mutant. This should be commented on and discussed. It should also be mentioned that the particular atxr5 atxr6 double mutant combination is hypomorphic and not null.

We have clarified these points in the revised manuscript.

7) Figure 6F shows that line A5.B1.3 which has a seed pigmentation phenotype has a 5 bp insertion in the tt4 gene. It is stated that this is due to transposon remobilisation but it is not clear if this is inferred as a likely imprecise excision, or whether they are able to show that a TE is present in this location in a progenitor plant?

The 5 bp insertion in TT4 resembled a footprint caused by CACTA remobilisation. A similar footprint is shown in Figure 6E. In the case of TTG4, we did not find the intact transposon in plants from previous generations.

8) Given that a lot of sequence data is available for some of the lines, I wondered if authors should check that there are no mutations present in genes associated with RdRM etc? That is not intended as a slight, but since changes in DNA methylation are observed that don't readily correlate with what is seen in the ref6 elf6 parental lines it could presumably be easily checked and eliminated as a potential cause.

We have investigated the chromatin (H3K27me3 and H3K27me1) and expression profile of gene implicated in DNA methylation genes, both in our histone demethylase mutants and also in epiERs (see heatmap in response to reviewer 2). We only found one differentially expressed gene (FDM2) that acts redundantly with five other genes (FDM1, FDM3, FDM4 and FDM5) in RNA-directed DNA methylation (Xie et al., 2012).

9) The correlation between pericentromeric H3K27me3 increase and DNA methylation decrease is an interesting observation, but they provide no mechanism why this should arise in elf6 ref6 backgrounds, which they show seem normal for pericentromeric DNA methylation and H3K27me3. The Qiu et al. (2019) Nat Comm paper, which the authors here mention in the Introduction, reported that REF6 cannot bind efficiently on methylated DNA, so it is puzzling.

Our data combined with recent work (Borg et al., 2020) supports the hypothesis that an increase in H3K27me3 levels in histone demethylase mutants causes defects on the epigenomic reprogramming that takes place during gametogenesis, primarily at pericentromeric regions.

10) Lines were picked based on aberrant phenotypes, propagated, and then analysed and found to contain various epigenetic and genetic changes. It is difficult to know whether the changes cause the phenotypes seen, and if so which particular changes. This made me wonder to what extent ELF6 REF6 F2 progeny with normal phenotypes would also show epigenomic changes, or whether this is specific to the plants with phenotypic abnormalities?

For practical reasons we did not perform epigenomic analyses in plants from ELF6 REF6 progenies that were indistinguishable from wild-type. We cannot exclude that “normal-looking” plants may contain a small number of epigenomic modifications, but this does not affect any of our conclusions.

11) The fact that ref6 and elf6 have opposite effects on flowering time is curious. The elf6c mutant is early flowering, consistent with a role for ELF6 in binding the floral repressor FLC and promoting its expression by H3K27me3 demethylation (Mol Plant 11:1135). The ref6 mutant is late flowering, and has been correlated with increased FLC expression (Plant Cell 16: 2601), which seems at odds with the hypermethylation for H3K27me3 and decreased expression more typical of these mutants. This might be commented on, and whether the various profiling experiments offer any explanation.

We agree that it would be interesting to investigate why *ref6-5* and *elf6-C* have opposing effects on flowering but in order to define the precise mechanism(s) it would be desirable to conduct a detailed analysis, which is beyond the scope of the current study.

[Editors' note: further revisions were suggested prior to acceptance, as described below.]

Reviewer #3:

The authors have provided some new data, ChIP seq of H3K27me1 in clf swn mutants, which provides some support for their model that euchromatic H3K27me1 derives from demethylation by REF6 of H3K27me3 deposited by PRC2 and is a good addition. Overall I find it an interesting paper of broad general interest but don't feel that the revision greatly addresses the comments from before as to mechanism and significance of H3K27me1 being unclear. That said, there is a lot of data in the paper and it is an interesting story that is perhaps complementary to the Borg et al., 2020 paper.

I have issues with a couple of the points made by authors.

3) Much of the data is not accessible or presented. For example, no lists of the peaks found for the different marks profiled are provided, nor are there lists of differential peaks found, or how these related to genes showing altered expression. All the raw data has been uploaded in a public repository. We don't think it is necessary to upload all of the peak data as supplemental information.

It is a given that the raw data will be provided. Since there is a lot of discussion of the ChIP seq data and selected peaks etc are shown I think it reasonable that the authors provide lists of enriched genes for the various ChIP seq experiments, as is usually provided in other papers.

We have included in the revised manuscript a table with a list of genes enriched for the different ChIP-seq experiments.

8) Given that a lot of sequence data is available for some of the lines, I wondered if authors should check that there are no mutations present in genes associated with RdRM etc? That is not intended as a slight, but since changes in DNA methylation are observed that don't readily correlate with what is seen in the ref6 elf6 parental lines it could presumably be easily checked and eliminated as a potential cause.

We have investigated the chromatin (H3K27me3 and H3K27me1) and expression profile of gene implicated in DNA methylation genes, both in our histone demethylase mutants and also in epiERs (see heatmap in response to reviewer 2). We only found one differentially expressed gene (FDM2) that acts redundantly with five other genes (FDM1,

This does not answer the question. A mutation in one of the RdRM genes will not necessarily alter expression level (nonsense mediated decay could occur, but not necessarily) or chromatin.

In our previous response, we provided evidence that the expression of RdDM genes is not affected in our histone demethylase mutants nor in the epimutants. We have also shown in our previous response that RdDM genes do not accumulate chromatin changes (H3K27me1 and H3K27me3) in these plants. We have conducted a thorough mutation analysis in our histone demethylase mutants and epiERs but we have found no evidence of mutations in any of the genes implicated with RdDM or other DNA methylation pathways.

In addition, the fact that some epiER plants display partial restoration of DNA methylation at transposons and pericentromeric chromatin regions (See Figure 3B-C for details) lends further support that the changes in DNA methylation observed in epiERs are not caused by mutations in RdDM genes.
